# The renaissance and enlightenment of *Marchantia* as a model system

**DOI:** 10.1093/plcell/koac219

**Published:** 2022-08-17

**Authors:** John L Bowman, Mario Arteaga-Vazquez, Frederic Berger, Liam N Briginshaw, Philip Carella, Adolfo Aguilar-Cruz, Kevin M Davies, Tom Dierschke, Liam Dolan, Ana E Dorantes-Acosta, Tom J Fisher, Eduardo Flores-Sandoval, Kazutaka Futagami, Kimitsune Ishizaki, Rubina Jibran, Takehiko Kanazawa, Hirotaka Kato, Takayuki Kohchi, Jonathan Levins, Shih-Shun Lin, Hirofumi Nakagami, Ryuichi Nishihama, Facundo Romani, Sebastian Schornack, Yasuhiro Tanizawa, Masayuki Tsuzuki, Takashi Ueda, Yuichiro Watanabe, Katsuyuki T Yamato, Sabine Zachgo

**Affiliations:** School of Biological Sciences, Monash University, Melbourne VIC 3800, Australia; ARC Centre of Excellence for Plant Success in Nature and Agriculture, Monash University, Melbourne VIC 3800, Australia; Instituto de Biotecnología y Ecología Aplicada, Universidad Veracruzana, Xalapa VER 91090, México; Gregor Mendel Institute (GMI), Austrian Academy of Sciences, Vienna Biocenter (VBC), Vienna 1030, Austria; School of Biological Sciences, Monash University, Melbourne VIC 3800, Australia; ARC Centre of Excellence for Plant Success in Nature and Agriculture, Monash University, Melbourne VIC 3800, Australia; Department of Cell and Developmental Biology, John Innes Centre, Norwich NR4 7UH, UK; Instituto de Biotecnología y Ecología Aplicada, Universidad Veracruzana, Xalapa VER 91090, México; The New Zealand Institute for Plant and Food Research Limited, Palmerston North 4442, New Zealand; School of Biological Sciences, Monash University, Melbourne VIC 3800, Australia; Gregor Mendel Institute (GMI), Austrian Academy of Sciences, Vienna Biocenter (VBC), Vienna 1030, Austria; Instituto de Biotecnología y Ecología Aplicada, Universidad Veracruzana, Xalapa VER 91090, México; School of Biological Sciences, Monash University, Melbourne VIC 3800, Australia; ARC Centre of Excellence for Plant Success in Nature and Agriculture, Monash University, Melbourne VIC 3800, Australia; School of Biological Sciences, Monash University, Melbourne VIC 3800, Australia; ARC Centre of Excellence for Plant Success in Nature and Agriculture, Monash University, Melbourne VIC 3800, Australia; Department of Life Sciences, Graduate School of Arts and Sciences, The University of Tokyo, Tokyo 153-8902, Japan; Graduate School of Science, Kobe University, Kobe 657-8501, Japan; The New Zealand Institute for Plant & Food Research Limited, Auckland 1142, New Zealand; Division of Cellular Dynamics, National Institute for Basic Biology, Myodaiji, Okazaki, Aichi 444-8585, Japan; The Department of Basic Biology, SOKENDAI (The Graduate University for Advanced Studies), Okazaki, Aichi 444-8585, Japan; Graduate School of Science, Kobe University, Kobe 657-8501, Japan; Graduate School of Science and Engineering, Ehime University, Matsuyama, Ehime 790-8577, Japan; Graduate School of Biostudies, Kyoto University, Kyoto 606-8502, Japan; School of Biological Sciences, Monash University, Melbourne VIC 3800, Australia; Institute of Biotechnology, National Taiwan University, Taipei 106, Taiwan; Basic Immune System of Plants, Max-Planck Institute for Plant Breeding Research, 50829 Cologne, Germany; Department of Applied Biological Science, Tokyo University of Science, Noda, Chiba 278-8510, Japan; Department of Plant Sciences, University of Cambridge, Cambridge CB2 3EA, UK; Sainsbury Laboratory, University of Cambridge, Cambridge CB2 1LR, UK; Department of Informatics, National Institute of Genetics, Mishima, Shizuoka 411-8540, Japan; Department of Life Sciences, Graduate School of Arts and Sciences, The University of Tokyo, Tokyo 153-8902, Japan; Division of Cellular Dynamics, National Institute for Basic Biology, Myodaiji, Okazaki, Aichi 444-8585, Japan; The Department of Basic Biology, SOKENDAI (The Graduate University for Advanced Studies), Okazaki, Aichi 444-8585, Japan; Department of Life Sciences, Graduate School of Arts and Sciences, The University of Tokyo, Tokyo 153-8902, Japan; Faculty of Biology-Oriented Science and Technology, Kindai University, Kinokawa, Wakayama 649-6493, Japan; Division of Botany, School of Biology and Chemistry, Osnabrück University, Osnabrück 49076, Germany

## Abstract

The liverwort *Marchantia polymorpha* has been utilized as a model for biological studies since the 18th century. In the past few decades, there has been a Renaissance in its utilization in genomic and genetic approaches to investigating physiological, developmental, and evolutionary aspects of land plant biology. The reasons for its adoption are similar to those of other genetic models, e.g. simple cultivation, ready access via its worldwide distribution, ease of crossing, facile genetics, and more recently, efficient transformation, genome editing, and genomic resources. The haploid gametophyte dominant life cycle of *M. polymorpha* is conducive to forward genetic approaches. The lack of ancient whole-genome duplications within liverworts facilitates reverse genetic approaches, and possibly related to this genomic stability, liverworts possess sex chromosomes that evolved in the ancestral liverwort. As a representative of one of the three bryophyte lineages, its phylogenetic position allows comparative approaches to provide insights into ancestral land plants. Given the karyotype and genome stability within liverworts, the resources developed for *M. polymorpha* have facilitated the development of related species as models for biological processes lacking in *M. polymorpha*.

## Teaser

The model liverwort Marchantia polymorpha is now experiencing a Renaissance due to its facile forward and reverse genetics and its simple genome that includes sex chromosomes.

## Introduction

During the recent revival of *Marchantia polymorpha* as a model system a number of reviews have covered the anatomical details of its life cycle (Shimamura, 2016; Bowman, 2022), the history of its use in experimental biology (Bowman, 2016), nomenclatural issues (Bowman et al., 2016), the development of genetic and genomic tools (Ishizaki et al., 2016; Nishihama et al., 2016), synthetic biology (Sauret-Güeto et al., 2020), and recent advances in understanding its physiology and development (Hisanaga et al., 2019b; Kohchi et al., 2021). Therefore, in this review, we complement the available literature by first providing a phylogenetic perspective on *Marchantia*, followed by updates on genetic and genomic resources presently available, and conclude with a few vignettes on biological questions for which *Marchantia* has recently contributed new insights.

## The phylogenetic context of *Marchantia*

### Why a liverwort?

**Figure 1:**
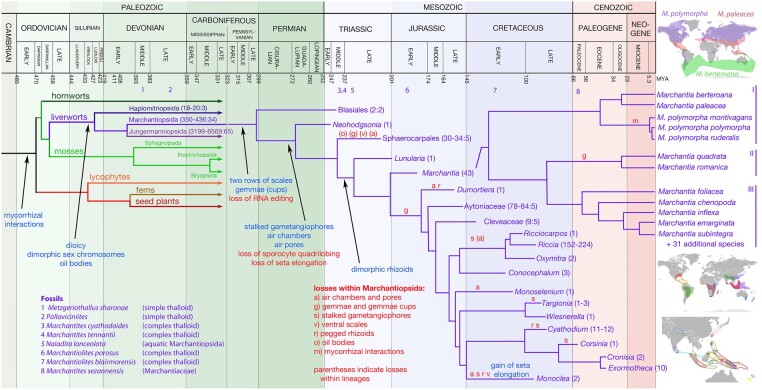
Phylogenetic context of *Marchantia*. The consensus phylogenetic history of *Marchantia* is plotted against the geologic timescale (top). The approximate divergence times of nodes in the trees were adapted from previous estimates of early land plant divergences (Morris et al., 2018) and nodes within the Marchantiopsida (Villarreal et al., 2016); Marchantiopsida phylogeny adapted from Villarreal et al. (2016). As there is substantial uncertainty in dating specific nodes, these should only be taken as an approximation. Further, the timing of the initial divergence between bryophytes and tracheophytes was adjusted to 470 MYA, the time of a shift in cryptospore morphology from irregular groupings (charophycean) to those typical of bryophytic meioses (i.e. land plant; Strother and Taylor, 2018). Previous estimates of divergence times (Villarreal et al., 2016; Morris et al., 2018) were calibrated with the fossil record. Just below the geologic time line are noted fossils relevant to Marchantiophyta evolution (e.g. Oostendorp, 1987; Townrow, 1958; Anderson, 1976; Tomescu et al., 2018; Lundblad, 1954; Brown and Robison, 1976; Hueber, 1961; Hernick et al., 2008; Harris, 1939; Kelber, 2019; de Saporta, 1868). Character evolution within the lineage leading to *Marchantia* is noted by origins (blue) and losses (red) of traits. Following taxa are numbers of extant species:genera; the lower numbers reflect accepted taxa (triple asterisks in Soderstrom et al., 2016) and the higher numbers include taxa not fully acknowledged (double asterisks in Soderstrom et al., 2016); numbers include accepted subspecies. In families with only a single genus, the genus name is provided rather than a higher order name. The three distinct *Marchantia* clades are denoted, I, II, and III. The approximate distributions of three clade I species are indicated in the top map. The lower two maps demarcate the distribution of clade III species, with each colour representing a different species, highlighting the diverse and restricted nature of their geographical distributions (Bischler, 1984; Bischler-Causse, 1989; Bischler-Causse, 1993).

Liverworts represent one of the ancient lineages of land plants (Embryophyta) that share a common ancestor, likely in the early-mid Ordovician. Currently accepted phylogenies and molecular clock-based dating calibrated with the fossil record (Morris et al., 2018; Leebens-Mack et al., 2019) suggest a rapid diversification with all three bryophyte lineages (hornworts, liverworts, mosses) and a vascular plant lineage being established in the Ordovician (Figure 1). The three vascular plant lineages (lycophytes, ferns, and seed plants) had diversified by the Early Devonian. Phylogeny reconstructions with most molecular datasets resolves bryophytes and tracheophytes as two sister monophyletic lineages (e.g. Li et al., 2020b; Leebens-Mack et al., 2019). However, ∼50% of gene trees fail to support the consensus tree topology presented in Figure 1 (Leebens-Mack et al., 2019), possibly due to the inferred rapid early diversification of extant lineages, especially those of the bryophytes which may have their independent origins in the Ordovician. As one of the six extant land plant lineages that have been evolving along independent trajectories since the Devonian (Figure 1), comparisons between liverworts and other lineages can inform us about the nature of ancestral versus derived characters; of particular interest are comparisons between bryophyte lineages and those of tracheophytes for inferences about the nature of the ancestral land plant (e.g. Bowman et al., 2019b). Such comparisons can reveal synapomorphies and derived features of Embryophyta as well as within the six extant land plant lineages. As any individual model species will be a combination of ancestrally inherited and derived characters, multiple models within each lineage are desirable to accommodate the deficiencies inherent in any one model.

### The relationship of *Marchantia* to other liverworts

The Marchantiophyta (liverworts) comprise three major extant lineages that diverged from one another during the Silurian to Devonian (Figure 1). Synapomorphies uniting liverworts include oil bodies, elaters, and dimorphic sex chromosomes (U, female; V, male) in dioicous species (Berrie, 1963; Crandall-Stotler et al., 2009), characters that have been investigated using *Marchantia* as a model (Yamato et al., 2007; Bowman et al., 2017; Romani et al., 2020; Kanazawa et al., 2020; Iwasaki et al., 2021). Three distinct liverwort gametophyte body plans exist: leafy shoots, simple thalloid forms with a uniform thallus structure, and complex thalloid forms that have distinct dorsal air chambers. The earliest diverging lineage in this group, the Haplomitriopsida, is species poor, consisting of three genera of leafy liverworts, with either an upright (*Haplomitrium*) or prostrate (*Treubia*, *Apotreubia*) habit, and when characterized, large genomes (Bainard et al., 2012). The other two lineages, the Marchantiopsida and Jungermanniopsida, likely diverged in the Devonian, and as the early diverging lineages of both have a simple thalloid gametophyte body plan, it may be assumed their common ancestor possessed one as well. The Marchantiopsida comprises 350–430 species in approximately 34 genera (Crandall-Stotler et al., 2009; Soderstrom et al., 2016), most of which have a complex thalloid gametophyte body plan or were derived from such an ancestor. The third lineage, the Jungermanniopsida, is the most species rich (4,000–7,000 species) and consists of lineages with a presumably ancestral simple thalloid gametophyte body plan, and lineages with a derived leafy gametophyte body plan. Much of the species diversity within the Jungermanniopsida has its origins in the Cenozoic (Cooper et al., 2012; Feldberg et al., 2014), although the families and orders obviously have older origins.

The genus *Marchantia* is nested within the Marchantiopsida, a class characterized by a few species-rich families and many species poor, sometimes monotypic, families (Figure 1). Several conspicuous attributes of *Marchantia*, especially with respect to its complex thalloid gametophyte body plan (reviewed in Shimamura, 2016; Bowman, 2022), asexual reproduction via multicellular discoid gemmae, and highly modified thalloid gametangiophores that elevate the sexual structures and subsequently the sporophyte for spore dispersal are derived characters within the Marchantiopsida (Figure 1). The presence of these features in early diverging Marchantiopsida lineages, e.g. *Neohodgsonia mirabilis*, indicates that the characters evolved early in the lineage and were subsequently lost in many derived taxa (Figure 1). The overall pattern of morphological evolution within the Marchantiopsida has been one of initial elaboration of the thallus and gametophores followed by a subsequent reduction in the complexity of these organs (Goebel, 1930). *Riccia* species provide a most conspicuous example, with both the gametophyte and sporophyte generations reduced from the ancestral condition. This pattern has been likened to neoteny whereby juvenile characters are retained in the adult and has been hypothesized to be an adaptation to colonization of increasingly variable and ephemeral habitats, in this case perhaps dating back to the Mesozoic radiation of derived Marchantiopsida (Schuster, 1992a).

Among the Marchantiopsida, genetic and genomic resources are being developed for a number of liverworts, and in this regard, the *Marchantia* genome can act as a Rosetta stone for the genomes of other Marchantiopsida species. *Riccia fluitans* and *Ricciocarpos natans* have independently evolved derived aquatic lifestyles and exhibit broad morphological plasticity in response to growth in terrestrial versus aquatic habitats. Single *R. fluitans* plants can consecutively adapt to the different growth environments, with differences in air chamber/pore and rhizoid development depending upon habitat (Althoff and Zachgo, 2020). In addition, secondary losses of pegged rhizoids and complex air pores in *R. fluitans* might have evolved in adaption to the amphibious life style. Transformation and sexual organ induction protocols have been established for *R. fluitans* (Althoff and Zachgo, 2020), and adaptation of the protocols developed for *Riccia*, in addition to those for *Marchantia* (see below for details), and could be attempted in other Marchantiopsida (and liverworts more broadly) to phylogenetically extend functional studies.

### *Marchantia polymorpha* within the genus *Marchantia*

The genus *Marchantia* consists of approximately 40 described species that resolve into three distinct clades (Figure 1), with species in each clade united by geographic distribution, morphology, and ecology (Bischler, 1984; Bischler-Causse, 1989; Bischler-Causse, 1993; Forrest et al., 2006; Long et al., 2016). Species within clade I have broad global latitudinal distributions. *Marchantia polymorpha*, which consists of three subspecies (see below), is widely distributed across the northern hemisphere in a largely Holoarctic realm. *Marchantia polymorpha* subsp. *ruderalis*, the subspecies used as a genetic model system, has been disseminated by humans to most inhabited regions of the world, including areas of human settlement in many places throughout the Southern hemisphere (Schuster, 1992b). In the corresponding latitudes in the southern hemisphere is *M. berteroana* with a Holoantarctic distribution including many subantarctic islands suggesting long distance dispersal is not limiting (Hooker, 1867; Longton and Holdgate, 1979). Its similarity in both morphology and distribution led Hooker to remark “This is the southern representative of the ubiquitous northern *M. polymorpha*, differing from that plant in the more convex fronds without a midrib, more minute cells, and more prominent pores” (Hooker, 1867). A third species of the clade, *M. paleacea*, has a circumglobal distribution between that of *M. polymorpha* and *M. berteroana*, being found at lower latitudes from Southeast Asia through the Mediterranean and the North American subtropics.

The two species of clade II, *M. quadrata* and *M. romanica*, are united by several morphological features including a four-lobed archegoniophore and lack of gemmae cups, with the lack of gemmae necessitating sexual reproduction in these species. Both species also have a pan-continental, albeit limited, distribution largely in the northern hemisphere. Enzyme polymorphism is detected in populations of *M. quadrata* from Europe, Asia and North Americas (Boisselier-Dubayle and Bischler, 1997) and rDNA sequences differ significantly between populations suggesting *M. quadrata* as circumscribed might represent multiple taxa. Indeed, two proposed subspecies based on sexual differences, dioicous versus monoicous have been proposed (Schuster, 1992b); however, no known genetic or other morphological markers correlate with these proposed subspecies. For a discussion of the sexuality of *M. quadrata* and other species of *Marchantia* that deviate from strict dioicy, see Bowman (2016).

The remaining species in the genus, those in clade III, have either regional, sometimes pan-continental, or local geographic distributions, with a large number of species in the Indo-Australian archipelago (Bischler, 1984; Bischler-Causse, 1989; Bischler-Causse, 1993). At first glance the concentration of species in the Indo-Australian archipelago might suggest a geographical origin of diversification, but the phylogeny of the genus does not support this hypothesis. It is more likely that *Marchantia* has undergone a (relatively) recent radiation in the Indo-Australian archipelago. Ascertaining their relationships via a molecular phylogeny is needed to test this hypothesis, with preliminary data linking these Indo-Australian archipelago species with central and South American species. Most species of clade III inhabit stable, moist to wet habitats, but they may also colonize relatively stable disturbed habitats, such as roadsides and trail margins. However, even the more widely distributed species do not appear to have become weedy. Thus, the species might be classified as perennial shuttle or perennial stayer species (During, 1979). Despite this, the existence of several species limited to specific Pacific islands (e.g. Hawaii, New Caledonia, Society Islands) indicates that species of this clade possess the capacity for long distant dispersal.

### *Marchantia polymorpha* subsp. *Ruderalis* within *M. polymorpha* sensu lato

*Marchantia polymorpha* is sometimes referred to as a species complex, consisting of three distinguishable subspecies that were acknowledged by Micheli (1729) prior to Linnaeus lumping them together under the moniker “polymorpha” (Linné and Salvius, 1753). The three subspecies are estimated to have diverged beginning about 5 million years ago (Villarreal et al., 2016). The three subspecies can be interbred, but reciprocal crosses were not always successful, e.g. female *M. polymorpha* subsp. *Montivagans* were successfully crossed with male *M. polymorpha* subsp. *Polymorpha*, but the reciprocal cross was infertile (Burgeff, 1943). Furthermore, evidence of interbreeding was not detected in sympatric populations of *M. polymorpha* subsp. *Polymorpha* and *M. polymorpha* subsp. *Ruderalis* within a few meters from one another in a streamside habitat (Boisselier-Dubayle et al., 1995). In such cases, cross-fertilization potential is not expected to be limited as fertilization within *M. polymorpha* subsp. *Ruderalis* has been observed at a distance up to 19 m in a meadow habitat (Pressel and Duckett, 2019). For a more detailed history of nomenclature and rationale for considering them as three species or three subspecies see Bowman et al. (2016). Recent analyses indicate that the three subspecies have unique chloroplast and mitochondrial haplotypes (Kijak et al., 2018; Linde et al., 2020), but that their nuclear genomes exhibit evidence of hybridization and introgression when subspecies are sympatric (Linde et al., 2020). However, based on examination of three to five individuals of each subspecies collected primarily from Sweden, only limited genomic regions were detected to be exchanged between subspecies in each hybridization event, with different genomic regions susceptible to varying levels of introgression (Linde et al., 2020). These data suggest the three lineages might be considered incipient species but their remarkable dispersal potential and sympatric habitats could lead to continued gene flow between lineages, especially for adaptive alleles for which selection acts in the haploid gametophyte (Linde et al., 2020). Genome analyses of plants of the three subspecies from a broader geographic sampling has potential to provide a detailed view of evolution within this species complex. In addition, such sampling would provide insight into the nature of the pangenomes of the three individual lineages and the overlap of the respective pangenomes. Collection of additional accessions from locations spanning the natural and man-made habitats of *M. polymorpha* would facilitate populations studies.

## Life cycle

**Figure 2:**
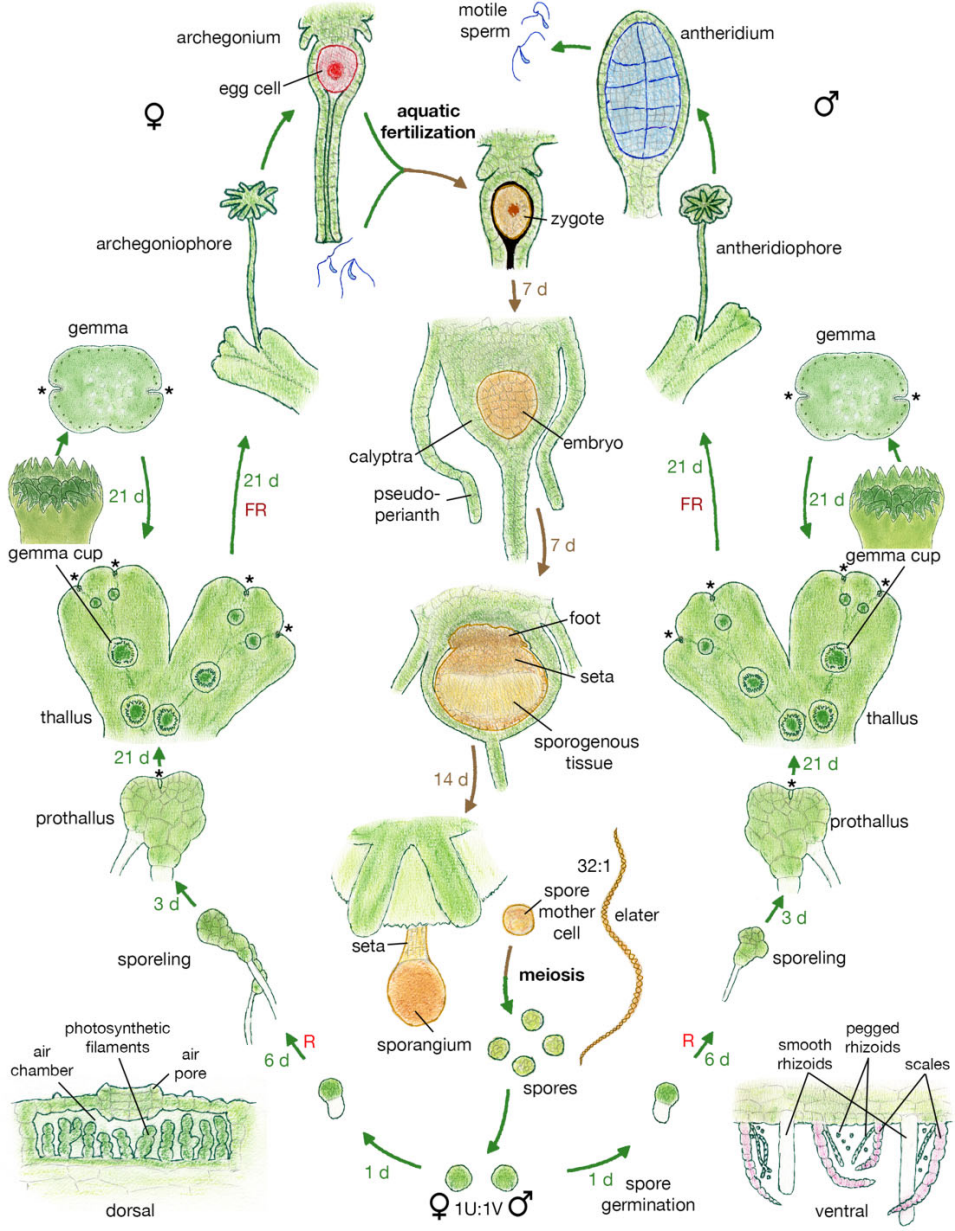
Life cycle of *M. polymorpha*. The haploid stages of the life cycle are depicted in shades of green, except for the female and male gametes; diploid stages of the life cycle are depicted in shades of brown. Gametophytic vegetative growth is largely indistinguishable between males and females, but this is not the case for all liverworts. Asterisks mark the apical meristems during the vegetative stages of the life cycle. Lower left and lower right show cross-sections of the dorsal (left) and ventral (right) regions of the complex thallus. Approximate times for the transitions between stages under optimal growth conditions is listed in days (d). R, red light; FR, far-red light. See text for more detailed description of life cycle stages. Some drawings were adapted from early literature: prothallus (Leitgeb, 1881), gemma and gemma cup (Mirbel, 1835), archegonium and antheridium (Strasburger et al., 1912), elater (Henfrey, 1853), air chamber (Kny, 1890); all other drawings by JLB.

*Marchantia* has a typical gametophyte dominant bryophytic life cycle, the anatomical details of which have been elucidated over the past couple centuries (Bowman, 2022), and the modern view has been recently reviewed (Shimamura, 2016), and the reader is referred to these reviews for references to earlier literature. Here we provide an overview focusing on attributes favorable for its use as a model genetic organism (Figure 2).

Dispersed haploid spores represent the initiation of the gametophyte stage of the life cycle, and their germination requires light. As *M. polymorpha* is dioicous with sex chromosomally determined, spores are formed with equal numbers of males with V chromosomes and females with U chromosomes. Sporeling development is variable and dependent upon environmental conditions (e.g. red light) and can be divided into two stages. The first cell division usual produces a rhizoid initial with the other cell developing into the remainder of the sporeling followed by subsequent cell divisions that produce an irregular mass of photosynthetic tissue along with (basal) rhizoids. Then, between 7 and 10 days of growth an apical cell with two cutting faces is specified, and subsequent development from this apical cell produces a 2D prothallus. Efficient genetic transformation using *Agrobacterium tumefaciens* (*Agrobacterium radiobacter*) is possible at the late sporeling stage, with the spores from a single sporophyte capsule potentially producing thousands of primary transformants (Ishizaki et al., 2008). Following a short growth phase as a prothallus, development transitions from 2D- to 3D growth, via a transition from two to four cutting faces of the apical cell within the shoot meristem. Three-dimensional growth enables production of specific differentiated tissues specialized for water and nutrient uptake ventrally (scales, two types of rhizoids, etc.) and photosynthesis dorsally (air chambers, gemma cups, etc.) typical of a complex thalloid liverwort. Unicellular smooth rhizoids both anchor the thallus to a substrate and mediate nutrient uptake from the substrate. In liverworts that form mycorrhizal interactions, the fungal infection is usually via the smooth rhizoids. Scales, often pigmented with auronidin, initially protect the shoot apex and subsequently act with associated unicellular pegged rhizoids (dead at maturity) to facilitate water distribution across the thallus. This growth phase is the typical one encountered in nature and can last for months to years.

Asexual propagation is achieved via multicellular lenticular gemmae formed from the base of gemmae cups, which are produced on the dorsal midline following apical bifurcation (branching) events occurring during the non-inductive conditions for sexual reproduction. The gemmae are dormant when within the cup, but when dispersed will develop into clonal independent thalli. When thalli with gemma cups harboring gemmae are gradually desiccated, the parental thallus will perish, but the gemmae remain viable, providing a medium to long approach for propagation of lines. Critically, as gemmae are derived from single cells at the gemma cup base they allow the clonal purification of mutations so as to avoid working with chimeric transformants (Ishizaki et al., 2016), providing an abundant source of clonal plants with a homogeneous developmental starting point ideal for experimental protocols. Gemmae are also a substrate amenable to *Agrobacterium*-mediated transformation (Tsuboyama and Kodama, 2018), a technique particularly useful for transformation of a uniform genetic background.

The “mature” vegetative thallus has a complex thalloid body plan which is derived from shoot apical meristems within notches at the growing tips (Leitgeb, 1881). In response to longer days and specifically and increased ratio of far-red (FR) to red light, thalli can be induced to begin sexual reproduction (Inoue et al., 2019). Following induction, one of the two branches from a bifurcation initiates a modified growth pattern producing structures called gametangiophores. The shoot meristem of each gametangiophore typically undergoes three successive dichotomous branching events, leading to an eight-lobe antheridiophore, where growth is mostly apical or a nine-lobed archegoniophore, where growth is primarily between apices. Gametangiophores vertically elevate the gametangia, such that when the maternally dependant sporophyte matures it is also elevated for more efficient spore dispersal. Archegonia for females and antheridia for males, are produced from dorsal derivatives of the shoot apical meristem such that many can develop from each apex (Leitgeb, 1881). A single egg cell develops at the base of each archegonium, with each antheridum producing many hundreds of motile sperm. The dioicous condition facilitates crossing, with antheridiophores producing mature sperm arising slightly prior to archegoniophores producing receptive eggs. However, unless inbred lines are established the dioicous condition also introduces polymorphisms since male and female plants collected from the same locality are often polymorphic (Bowman et al., 2017; Montgomery et al., 2020).

Aquatic fertilization produces the zygote, which is enclosed in two maternal gametophytic tissues, the calyptra, derived from the archegonium, and another layer, the pseudoperianth. Following the formation of the zygote, the sporophyte undergoes dispersed cell division to produce a multicellular embryo, but no apical meristem (or cell) is evident during sporophyte development (Kienitz-Gerloff, 1874). The early embryo appears relatively uniform, but by 2-week post fertilization three distinct tissues: foot, seta, and capsule, which house the sporogenous tissue, are evident. The foot has intimate connection with the maternal gametophyte, and through this placental connection nutrients and water are supplied from the gametophyte to the sporophyte. The capsule, or sporangium, has a unistratose wall, except at its apex, and within the sporogenous tissue two cell types differentiate: spore mother cells and elaters, at a ratio of approximately 32:1. The spore mother cells undergo meiosis to produce up to 300,000 spores per capsule (O'Hanlon, 1926). The elater cells, which undergo programmed cell death, produce helical wall thickenings that via hygroscopic movements mechanically aid in spore dispersal. During the majority of its development, the sporophyte is surrounded by gametophytic tissue, with the calyptra, derived from the archegonium wall, being in intimate association with the sporophyte. Once the capsule is mature, the seta elongates by cell elongation pushing the capsule free of maternal tissues and rupture of the capsule wall allows spore dispersal. Detailed descriptions of each of the life stages and references to the earlier literature can be found in recent reviews (Shimamura, 2016; Bowman, 2022).

## Ecology of *M. polymorpha*

Two species of clade I, *M. polymorpha* and *M. berteroana*, can be classified as having a colonist life strategy, with long-distance colonization most often via sexually produced spores, but sometimes via gemmae, with gemmae contributing to extensive colony growth following establishment. The widespread dissemination of spores exemplified by the recovery of a few hundred *M. polymorpha* spores from 1,373 mL of rainwater collected at the Zoological Station at Tviirminne, Finland in the summer of 1936 (Pettersson, 1940). As mentioned previously, *M. polymorpha* is well known as a weedy colonizer of disturbed, often man-made, habitats (Schuster, 1992b). Both *M. polymorpha* in the northern hemisphere and *M. berteroana* in southeast Australia are rapid colonizers, often on bare ground, following fire (e.g. Hooker, 1860; Kelly, 1914; Skutch, 1929; Torrey, 1932; Crane et al., 1983; Brasell and Mattay, 1984). Due to its ability to colonize barren soils, *M. polymorpha* was tested for its ability to colonize lunar soils following the Apollo missions, and it thrived on these substrates (Walkinshaw et al., 1970). The loss of the ability to interact with mycorrhizal fungi in *M. polymorpha* may be related to the ecological adaptation to rapid colonization and is an evolutionarily recent event, postdating the divergence of *M. polymorpha* from the other clade I species (Ligrone et al., 2007; Radhakrishnan et al., 2020). Perhaps in compensation for this loss, gene families encoding phosphate and ammonium transporters are among those overrepresented in the *M. polymorpha* genome (Bowman et al., 2017).

### Interactions with the microbial world

The fossil record demonstrates that filamentous microbes invaded ancient plant cells with intracellular hyphal structures over 400 million years ago (Strullu-Derrien et al., 2014; Strullu-Derrien, 2018). Since then, land plants have evolved into a number of distinct lineages that continue to engage in interactions with detrimental and beneficial microbes. While research on plant–microbe interactions has historically focused on a limited number of angiosperms, the recent shift in focus toward non-standard and evolutionarily insightful land plants has resulted in the emergence of the evo-MPMI (evolutionary molecular plant–microbe interactions) field (Upson et al., 2018). Here, *Marchantia* shows potential to become an informative model for ecological surveys of liverwort-colonizing microbes in natural settings and for the molecular genetic dissection of plant immune processes in the laboratory.

Ecologically relevant associations between diverse microbial species and *Marchantia* have been investigated to varying degrees of specificity in distinct geographical regions. Initial surveys for endophytic (living within host tissues) and epiphytic (living on host surfaces) bacteria have been conducted in *M. polymorpha*, *M. paleacea*, and *M. inflexa* by amplicon sequencing of the bacterial 16S rRNA gene (Alcaraz et al., 2018; Marks et al., 2018). For *M. polymorpha* and *M. paleacea,* microbiomes of field-collected specimens (Veracruz, Mexico) were compared against the bacterial profiles of the surrounding soil, revealing associations with *Rhizobia* and *Methylobacterium* alongside an array of saprotrophic species thought to degrade dead or dying organic matter (Alcaraz et al., 2018). Associations with *Rhizobia* sp. were also observed in the microbiomes of *M. inflexa* collected from Trinidad and in specimens grown under greenhouse conditions in Kentucky (USA), which also revealed likely associations with Xanthomonads, Actinomycetes, and Caulobacter among others (Marks et al., 2018).

Comprehensive ecological surveys have defined the range of fungal species associated with liverworts through a combination of fungal amplicon sequencing and culturing methods that directly isolate endophytes for further experimentation. Massive sequencing surveys for fungal 18S rRNA within host tissues determined that several liverworts of the *Marchantia* genus were colonized by a diverse array of fungal endophytes belonging to the Claroideoglomeraceae, Diversisporaceae, Archaeosporaceae, and Glomeraceae (Rimington et al., 2018). Importantly, this revealed that *Marchantia* and other liverworts interact with distantly related lineages of arbuscular mycorrhizal fungi, with the exception of *M. polymorpha* and other nonsymbiotic species*. Marchantia polymorpha* interacts with a diverse set of fungal species with varying impacts on host fitness. Good examples of this are the pathogenic fungus *Irpex lacteus* (Matsui et al., 2020) and the basidiomycete *Loreleia marchantiae*, which predominantly colonizes rhizoids and thalli of the liverwort, causes little to no necrotic tissues, and forms attractive orange fruiting bodies (Bresinsky and Schötz, 2006). Additional interactions were resolved by Nelson et al. (2018), who employed culturing-based approaches in a range of *M. polymorpha* tissues (rhizoids, thalli, and gametophores) sampled across distant field sites in North America (USA and Canada). Laboratory based re-introduction assays combining individual fungal endophytes and *M. polymorpha* demonstrated that most strains had little impact on liverwort growth, with the exception of two species displaying strong growth promoting activity (*Nemania serpens* and *Colletotrichum truncatum*) and two others causing significant growth inhibition (*Xylaria cubensis* and *Hypoxylon submonticulosum*) (Nelson et al., 2018). Nelson and Shaw (2019) expanded on this search by performing molecular surveys in addition to culture-based isolation assays on diverse *M. polymorpha* subspecies in United States and Canada. Natural populations of *Marchantia* harbored diverse sets of fungal endophytes that varied between local populations of liverworts.

### Mycorrhizal interactions

While *M. polymorpha* does not support mycorrhizal fungal interactions, the other species of clade I, *M. berteroana* and *M. paleacea*, form mycorrhizal associations with Glomeromycotina fungi (Stahl, 1949; Ligrone et al., 2007; Humphreys et al., 2010). Furthermore, liverworts as a whole have been shown to form associations with all known orders of arbuscular mycorrhizal fungi (Rimington et al., 2018). *Marchantia paleacea* has been developed into an excellent model for such studies, with both a genomic sequence and transformation protocols available (Radhakrishnan et al., 2020; Rich et al., 2021). *Marchantia paleacea* occupies more stable moist habitats, such as streambanks, rock crevices, and edges of paths and road cuttings, with this “perennial stayer” life cycle strategy (During, 1979) consistent with the retention of the ancestral condition of forming mycorrhizal fungal associations. Previous comparative analyses have facilitated the identification of a suite of genes involved in the communications pathways between host and arbuscular mycorrhizal fungi required to activate the symbiotic program, as well as genes involved in the transfer of lipids from the host to the fungi (Radhakrishnan et al., 2020). Consistent with their abilities to form mycorrhizal associations, several genes required for the initial signaling events and establishment of a symbiotic interaction are present in the *M. paleacea* genome and lacking in the *M. polymorpha* subsp. *ruderalis* genome (Bowman et al., 2017, Radhakrishnan et al., 2020). Analyses of the genomes of the three *M. polymorpha* subspecies revealed that pseudogenes are detectable for four of six genes investigated, consistent with the loss being evolutionarily recent (Radhakrishnan et al., 2020).

Mycorrhizal fungi obtain carbon, including lipids, from their hosts. Using *M. paleacea* as a host for the arbuscular mycorrhizal fungus *Rhizophagus irregularis*, it was demonstrated that host derived lipids were transferred to the fungus, substantiating the mutualistic nature of their interaction (Rich et al., 2021). Furthermore, an AP2 transcription factor, Mpa*WRINKLED*, is necessary for successful establishment of arbuscular mycorrhizal associations, and that it is sufficient to activate fatty acid biosynthesis and lipid transfer gene expression (Rich et al., 2021). Since orthologous WRINKLED genes in angiosperms perform similar functions, the ancestral land plant was equipped with the genetic machinery required for establishment of mutualistic arbuscular mycorrhizal associations, a key innovation facilitating the evolution of land plants.

### Interactions with potential pathogens

The emerging ecological data have revealed diverse and complex relationships between *Marchantia* and its associated microbial communities, providing important natural context for comparative plant-microbe interactions research. While it is clear that further investigations are needed to fully appreciate the breadth of liverwort–pathogen interactions in nature, the emerging field of evo-MPMI research has adopted *Marchantia* as an informative platform to understand the evolutionary principles underpinning plant immunity and infection. Since much of our current understanding of plant defense originates from angiosperm model systems and crops, the dissection of plant–pathogen interactions in nonvascular plants is bound to uncover evolutionary innovations as well as conserved principles underlying plant immunity.

*Marchantia*–pathogen interaction research has thus far focused on hemi-biotrophic pathogens that manipulate living hosts before actively causing necrosis and subsisting on dead tissues. This is exemplified by the development of severe disease symptoms (chlorosis and/or necrotic lesions) on liverwort thalli upon infection with the oomycete *Phytophthora palmivora*, the bacterium *Pseudomonas syringae*, or the fungus *Fusarium oxysporum* (Carella et al., 2018; Gimenez-Ibanez et al., 2019; Redkar et al., 2022). In both instances, the colonization of liverwort thalli primarily occurs within the air chambers of the photosynthetic tissue layer. Current data suggest that air chambers likely act as an architectural susceptibility factor in liverworts, as *M. polymorpha nop1* mutants impaired in chamber development are more resistant to *P. palmivora*, *P. syringae,* or *Agrobacterium tumefaciens* infection (Carella et al., 2018; Iwakawa et al., 2021). In contrast, the storage tissue layer supports colonization by AM fungi in *M. paleacea* via the rhizoid entry route, which has not been observed for pathogens but is likely to occur. The successful infection of *M. polymorpha* thalli is also associated with pathogen virulence factor deployment. For *P. palmivora,* this was demonstrated by the specific *in planta* upregulation of known oomycete virulence factors (RxLR effectors, proteases, cell wall-degrading enzymes) and the development of intracellular infection structures (haustoria) recruiting host trafficking machinery (Carella et al., 2018). In the *P. syringae* pathosystem, mutants unable to translocate type-III secreted effector proteins (hrcC-) showed strong virulence defects in *M. polymorpha,* with further analyses identifying the effectors avrPto/avrPtoB as significant contributors to immune suppression in liverworts (Gimenez-Ibanez et al., 2019).

In comparison to flowering plants, where we have an extensive understanding of the molecular mechanisms underpinning plant immunity, much less is known in *Marchantia*. To date, emerging *Marchantia*–pathogen interaction research is beginning to clarify how liverworts respond to host invasion by pathogens. Liverworts demonstrate defense marker gene expression in response to molecular signatures delivered by pathogens (*P. syringae*, *F. oxysporum*), or through crude extracts containing microbe-associated molecular patterns (MAMP) epitopes likely detected by cell surface pattern recognition receptors, but these remain to be investigated (Gimenez-Ibanez et al., 2019; Redkar et al., 2022). Moreover, contributions of the major defense-related phytohormones, salicylic acid and jasmonate, to defend against bacteria and fungi were shown to resemble systems in angiosperms by the use of OPDA signaling deficient *M. polymorpha coi1* mutants or exogenous application of salicylic acid or jasmonate (OPDA) to the Tak-1 *M. polymorpha* accession (Gimenez-Ibanez et al., 2019; Matsui et al., 2020; Iwakawa et al., 2021). Comparisons between liverwort (*M. polymorpha)* and angiosperm (*Nicotiana benthamiana*) transcriptomes during *P. palmivora* infections identified common plant defense responses centred on pathogenesis-related (PR) proteins and phenylpropanoid metabolism (Carella et al., 2019). Further analysis demonstrated the importance of the MYB transcription factor MpMyb14 in regulating phenylpropanoid biosynthesis during infection, with Mp*myb14* mutants having enhanced susceptibility to *P. palmivora* infection, whereas the overaccumulation of MpMyb14-dependent phenylpropanoids and pigments provided significant resistance (Carella et al., 2019). Collectively, these studies demonstrate the utility of *Marchantia* as an accessible experimental system to interrogate the conserved and divergent aspects of plant immunity relevant to agriculturally relevant pathogens like *Phytophthora* or *Pseudomonas.* Looking forward, *Marchantia* and other nonvascular models provide unprecedented potential for the discovery of conserved mechanisms underpinning plant immune responses and the identification of lineage-specific disease resistance strategies that can be leveraged for biotechnological application.

## *Marchantia* Genomics

### Nuclear genome of *M. polymorpha*

A female line of *M. polymorpha* subsp. *ruderalis*, which had been obtained by backcross between Tak-1 and Tak-2 and thus has the autosomes mostly from Tak-1 and the U (female) sex chromosome from Tak-2, was used to generate the reference genome, with the first assembly 3.1 based on short read sequences (Bowman et al., 2017). The subsequent v6.1 assembly by pseudo-chromosome was obtained using HiC on a combination of short and long read sequences and gene nomenclature was updated following the conventions used for *Arabidopsis thaliana* (Montgomery et al., 2020; Iwasaki et al., 2021).

The *M. polymorpha* genome is about 220-Mb distributed in nine chromosomes that contain approximately 20,000 protein coding genes and 30% transposons and repeats (Bowman et al., 2017; Montgomery et al., 2020). Analyses of the genomes of *M. polymorpha* and other model bryophytes, such as the moss *Physcomitrium patens* and the hornwort *Anthoceros agrestis*, show that whole genome duplications (WGD) occurred in mosses but is absent in *Marchantia* and *Anthoceros* (Bowman et al., 2017; Lang et al., 2018; Li et al., 2020b). These genomes also share the same striking alternation of protein coding genes, transposons, and repeats (Bowman et al., 2017; Lang et al., 2018; Montgomery et al., 2020; Li et al., 2020b). This bryophyte-specific organization of the genome is mirrored with the presence of clear Topologically Associating Domains (TADs) which are delimited by plant-specific class 1 transcription factor TCP1 also found at the border of TADs in rice (Liu et al., 2017). Unlike rice TADs that contain genes, *Marchantia* TADs contain transposons and repeats and are associated with heterochromatic modifications (Karaaslan et al., 2020).

The accumulation of transposons and repeats around centromeres in genomes of angiosperms often lead to a clear separation of heterochromatin into distinct nuclear domains termed chromocenters (Fransz et al., 2002; Chodavarapu et al., 2010). This organization contrasts with bryophyte genomes that do not organize heterochromatin into chromocenters (Heitz, 1929)—of note is that the original description of heterochromatin was in a Jungermanniopsida liverwort genus, *Pellia* (Heitz, 1928a; Heitz, 1928b). *Marchantia polymorpha* centromeres are bordered by enrichment in a specific family of LINE transposons (Montgomery et al., 2020). Another outstanding feature of *M. polymorpha* heterochromatin is the presence of the epigenetic mark H3K27me3 over circa half of transposons (Montgomery et al., 2020), whereas the other transposons are covered by epigenetic marks H3K9me1/2 and H3K27me1 found at heterochromatin of angiosperms (Sequeira-Mendes et al., 2014). In angiosperms H3K27me3 is deposited by the Polycomb Repressive Complex 2 (PRC2) and associated with repression of protein coding genes (Sequeira-Mendes et al., 2014; Schuettengruber et al., 2017). Yet H3K27me3 marks transposons targeted by DNA methylation when this DNA modification is defective (Deleris et al., 2012; Reddington et al., 2013; Saksouk et al., 2014; Walter et al., 2016). Since levels of DNA methylation in bryophytes are lower than in angiosperms and that specific DNA methylation pathways found in angiosperms are not present in bryophytes (Zemach and Zilberman, 2010; Takuno et al., 2016; Ikeda et al., 2018; Yaari et al., 2019), it is reasonable to propose that PRC2 was repressing transposons prior to DNA methylation in ancestors of land plants and that this role has been retained by liverworts. This is not the case in *P. patens* (Lang et al., 2018) and the presence of H3K27me3 on transposons in hornworts and charophycean algae remains to be established to get deeper insight in the potential ancestral role of this mark. The fluctuation of profiles of histone modifications during the life cycles of bryophyte is not known. However, amounts of DNA methylation at transposons and repeats fluctuates with a marked increased during gametogenesis and after fertilization (Schmid et al., 2018) but the mechanisms involved and the biological significance of this epigenetic reprograming event are unknown (Brautigam and Cronk, 2018). In *P. patens*, maintenance CG and de novo DNA methylation in CHH context depend on the ancestral DNMT1 and DNMT3 activities, respectively, that are conserved in most other eukaryotic lineages (Yaari et al., 2019). CHG methylation is deposited by plant-specific CHROMOMETHYLTRANSFERASEs (CMTs). It is proposed that early in angiosperm evolution the activity of DNMT3 was lost while the DNA methylation in CHH context became under the control of the siRNA-dependent de novo methylation pathway (RdDM), and a specialized form of CMT (Yaari et al., 2019). Understanding of the conservation of the activity of DNA methyltransferases in bryophytes requires further data from hornworts and liverworts to gain insight in the ancestral roles of these pathways.

Compared with charophycean algae, *M. polymorpha* chromatin diversity is also enhanced by evolution of a bryophyte-specific class of histone variants—H2A.M in addition to replicative H2A, H2A.Z and H2A.X shared with other plants (Kawashima et al., 2015). Bryophytes, including *M. polymorpha* also diversified the range of H2B variants while their genome encodes only the three types of H3 variants shared amongst most multicellular eukaryotes: cenH3 that defines the centromere and kinetochore function, replicative H3 expressed at S phase, and replacement H3.3 (Bowman et al., 2017; Jiang et al., 2020). Studies of the evolution of genes encoding the enzymes that write and erase the chromatin post-transcriptional modifications and their profiles in charophytes, bryophytes, and ferns are still too fragmentary (Bowman et al., 2017; Lang et al., 2018) to draw a global picture of evolution of the epigenome in land plants. Generating this data will be one of the next goals in the field together with gathering genomes of additional species at important positions in the land plant evolutionary tree.

### Sex chromosomes

Sex chromosomes in plants were first described in *Sphaerocarpos donnellii*, a member of the Marchantiopsida (Figure 1), with the female possessing a U chromosome (X in older literature) and the male a V (Y) chromosome (Allen, 1917). Almost a century later, the sequences of the genic regions of the *M. polymorpha* V chromosome provided the first glimpse into the structure of the sex chromosomes and their antiquity, given mutational saturation of third positions of codons for some presumptive U gametologs (Yamato et al., 2007). In the intervening decades, mutational experiments in *Sphaerocarpos*, *Marchantia*, and *Pellia* species wherein females could be converted into males, but not the converse, led to the idea of a dominant U-linked “feminizer” locus (Haupt, 1932; Knapp, 1935; Lorbeer, 1936; Lorbeer, 1938; Heitz, 1949). That ultraviolet (UV) aneuploids were female supports this hypothesis. In addition, these genetics experiments suggested that there were “motility” loci on the V chromosome of *Sphaerocarpos*, as the transformed males possessing a U chromosome produced sperm that was immotile (Knapp, 1936; Lorbeer, 1938).

In the current *M. polymorpha* assembly, v6.1, the genic regions of the U and V harbor 47 genes in 4.5 Mb and 93 genes in 7.5 Mb, respectively—a gene density about 20% that of autosomal chromosomes (Montgomery et al., 2020; Iwasaki et al., 2021). The lower gene density is consistent with degradation being of equal magnitude on the U and V chromosomes as segments became successively incorporated into non-recombining regions. The V chromosome encodes several proteins associated with flagellar function, making these candidates for the genetically defined “motility” loci (Bowman et al., 2017). The U and V chromosomes also share 19 pairs of gametologs, homologs descended from essential genes on the ancestral autosome that evolved into the sex chromosomes (Bowman et al., 2017). The divergence between the two gametologs has been used to map the antiquity of the non-recombining regions of the *M. polymorpha* U and V chromosomes, defining at least five evolutionary strata (Iwasaki et al., 2021). The oldest evolutionary stratum comprises 7 gametolog pairs that predate the divergence of extant liverwort lineages in the mid-Silurian 430MYA (Figure 1), indicating that the ancestral liverwort possessed sex chromosomes consistent with earlier karyotype analyses (Berrie, 1963; Iwasaki et al., 2021). Thus, the liverwort sex chromosomes evolved earlier than those known in any other eukaryote. Further, the identification of ancient gametologs provides a set of excellent candidates for sex-specific markers in any dioicious liverwort species.

One notable feature of the *M. polymorpha* U and V chromosomes is that there is no conspicuous pseudoautosomal region for pairing during meiosis. Indeed, there exists no synteny across the entire length of the genic regions *M. polymorpha* U and V chromosomes, with the entirely of this region predicted to be non-recombining. Of note are the extensive rDNA tracts present at both ends of the U chromosome and one end of the V chromosome (Nakayama et al., 2001; Fujisawa et al., 2003; Montgomery et al., 2020; Iwasaki et al., 2021). The other end of the V chromosome consists of a V-chromosome specific repeat sequences (Yamato et al., 2007; Montgomery et al., 2020). One possibility is that the rDNA tracts act in some manner as a pseudoautosomal region. The reported transient pairing and early dissociation (compared to autosomal pairs) of the *M. polymorpha* U and V during meiosis I (Haupt, 1932) might be a reflection of a non-conventional pseudoautosomal region. Meiotic pairing via rDNA repeats would not be unprecedented, as rDNA repeats mediate meiotic pairing between the X and Y sex chromosomes in *Drosophila melanogaster* (McKee and Karpen, 1990).

The availability of the U chromosome sequence facilitated the identification of the feminizer locus in *M. polymorpha* as *BASIC PENTACYSTEINE ON THE U CHROMOSOME* (Mp*BPCU*), encoding a transcription factor of a plant specific family (Iwasaki et al., 2021). Consistent with the earlier genetics, loss-of-function Mp*bpcU* alleles result in a female-to-male transformation and introduction of a wild-type copy of Mp*BPCU* into a male is sufficient for its transformation to a female morphology, albeit with non-motile sperm (Iwasaki et al., 2021). The target of MpBPCU is an autosomal sex-determining module consisting of a Myb transcription factor, *FEMALE GAMETOPHYTE MYB* (*FGMYB*), and its antisense long non-coding RNA, *SUPPRESSOR OF FEMINIZATION* (*SUF*) (Hisanaga et al., 2019a). In males, the transcription of *SUF* suppresses the expression of *FGMYB* resulting in initiation of male development. In contrast, in females, MpBPCU suppresses the transcription of *SUF*, resulting in the expression of *FGMYB* and initiation of female development (Iwasaki et al., 2021). Surprisingly, Mp*BPCU* has a V chromosome gametolog, Mp*BPCV*, with both genes required for the transition to reproductive growth in females and males, respectively, but with Mp*BPCV* lacking any sex determining activity (Iwasaki et al., 2021). This is in contrast with sex-determining genes in other characterized UV systems, where the sex-determining gene is usually a sex-specific gene, and opens questions as to whether other liverworts share BPCU as the feminizer and whether other dioicous bryophytes will possess a similar sex-determining system.

### Organellar genomes

The organellar genomes of *Marchantia* were among the first in land plants to be sequenced. The sequences of the complete chloroplast and mitochondrial genomes of *Marchantia paleacea* were determined by the classical dideoxynucleotide method (Ohyama et al., 1986; Oda et al., 1992). Note that these two genomes were originally published as those of *M. polymorpha*, but were later shown to be derived from *M. paleacea* cultures, with those of the *M. polymorpha* subsp. *ruderalis* published later with the nuclear genome (Bowman et al., 2017). Comparison with the organellar genomes of the other *M. polymorpha* subspecies supports the idea that the three subspecies had independent evolutionary trajectories in the past few million years (Linde et al., 2020). No RNA editing is present in *Marchantia* organelles due to the secondary loss in Marchantiopsida (Rüdiger et al., 2008). *Marchantia* is one of the few plant species where a plastid transformation method has been established (see “Reverse Genetic Approaches”), and thus has become a promising test-bed for chloroplast engineering, such as for protein hyperexpression (see “Synthetic Biology”).

While the structure and size of plastid genomes are largely conserved among land plants, including *M. polymorpha*, those of mitochondrial genomes are more divergent. The mitochondrial genomes of angiosperms generally consist of multiple DNA species, either generated by homologous recombination between repeat sequences (multipartite) or composed of separate chromosomes (multichromosomal; Sloan, 2013). In contrast, the mitochondrial genomes of bryophytes *M. polymorpha* and *P. patens* are represented by a single circular DNA (Oda et al., 1992; Terasawa et al., 2007). The biological significance and evolutionary origin of the multichromosomal system incorporating recombination of angiosperm mitochondrial genomes are yet to be elucidated.

### Resources and databases

Most of the intensively studied model organisms have dedicated genome databases. They are focused on a specific organism and play a pivotal role as a datahub and a knowledge base for genome-centric studies. Notable examples of this kind of genome database include The Arabidopsis Information Resource (TAIR) for *Arabidopsis thaliana* and the Saccharomyces Genome Database (SGD) for baker's yeast. As for *Marchantia*, MarpolBase is developed to provide genome and gene resources to the research community. It is equipped with a genome browser, keyword/sequence similarity search system, various analysis and utility tools, as well as gene nomenclature registration system.

At the time of writing this article, the latest version of the *M. polymorpha* genome sequence available at MarpolBase is v6.1 (MpTak_v6.1), which was constructed by combining the male and female reference genomes derived from *M. polymorpha* subsp. *ruderalis* Tak-1 and Tak-2, respectively. The autosomal and V chromosomal sequences are based on the male Tak-1 genome (MpTak1_v5.1), and the U chromosomal sequence is based on the female Tak-2 genome (MpTak2_v1.0), both of which were assembled using the PacBio long-read sequencing and the Hi-C scaffolding (Montgomery et al., 2020, Iwasaki et al., 2021). MpTak_v6.1 is meant for use as a standard reference in analyses that do not need to take sex differences into account. For the analyses specific to one of the sexes, it is advisable to use MpTak1_v5.1 or MpTak2_v1.0. The latest version of the gene annotation is revision 1 (MpTak_v6.1r1). In case of future updates, it is advised to keep track of which version of the genome/annotation you use in your research. As of v6.1, a gene locus identifier system similar to that for *Arabidopsis* has been introduced (e.g. Mp3g23300), where the third letter represents the chromosome number (1–8 for autosomes, U/V for sex chromosomes, or z for unplaced scaffold) and the trailing five-digit code represents relative position in the chromosome. The locus identifiers will be carried over as much as possible during any future genome or annotation updates. Transcript variants are distinguished by a transcript number following a period (e.g. Mp2g22820.1, Mp2g22820.2, …). In addition to the genomes mentioned above, MarpolBase provides three other *Marchantia* genomes, JGI v3.1: the previous version of the reference genome [*M. polymorpha* subsp. *ruderalis* Tak-1 BC4, (Bowman et al., 2017)]; MppBR5: *M. polymorpha* subsp. *polymorpha* strain BR5 (Linde et al., 2020); MpmSA2: *M. polymorpha* subsp. *montivagans* strain SA2 (Linde et al., 2020). The gene IDs of the v6.1 and v3.1 genomes are interchangeable using the converter available at MarpolBase. The plastid and mitochondrial genome data for Kitashirakawa-2 strain are also available for download. Other genome assemblies of *M. polymorpha* subsp. *ruderalis* have been published (Diop et al., 2020).

The Marchantia Gene Nomenclature DB hosted at MarpolBase (https://marchantia.info/nomenclature/), aims to provide a consistent and organized nomenclature system for *Marchantia* genes. To avoid redundancy and confusion in the scientific literature, genes should be named uniquely following the naming guidelines of Bowman et al. (2016), and it is strongly recommended that gene symbols and names should be registered to the database before publications or presentations. Registrations can be completed online, and users can keep the details confidential until the paper is published.

Genomes of other commonly used accessions Tak-2, Cam-1 and Cam-2, BoGa and the genome of *Marchantia paleacea*, which will be important to gain insight in evolution of symbiosis that is present in this species (Radhakrishnan et al., 2020) are also expected to be deposited at MarpolBase.

Apart from the organism-specific genome databases described above, there are many genome data resources available. The first place to visit is the public sequence databases of the International Nucleotide Sequence Database Collaboration (INSDC), which is jointly maintained by DDBJ in Japan, EMBL-EBI in Europe, and NCBI in the United States (Arita et al., 2021). Data deposition of the newly determined nucleotide sequences to the INSDC is a prerequisite for publication in many of the major scientific journals. Therefore, the databases of INSDC serves as a primary archival database that collects sequence data directly from researchers. INSDC covers a wide range of data types from raw sequencing reads to assembled genome sequences. The INSDC Assembly database is useful to search assembled genome sequences. However, by the nature of the archival database, annotation might be outdated or missing, and hence it is recommended to obtain the latest data from organism-specific databases if available. Sequence Read Archive (SRA) is a collection of raw sequencing read data, in which data are categorized depending on study type such as whole-genome sequencing or transcriptome sequencing. Reanalysis of the data obtained from INSDC is freely granted as long as appropriate credit is given by citing the original submission.

Phytozome and Ensembl Plants are both portal sites for assembled plant genomes. Phytozome mainly hosts genome data generated by the Joint Genome Institute (JGI) as well as for selected organisms sequenced by other parties. The previous version of the *M. polymorpha* reference genome (v3.1) was originally assembled and annotated by JGI. Data are available for download to registered user. Ensembl Plants imports genome assemblies from INSDC and gene models from either INSDC or other resources. The genomic data for *Marchantia* currently provided at Ensembl Plants is based on the v3.1 genome. Therefore, the genome sequences and gene models for *M. polymorpha* available from both Phytozome and Ensembl Plants are identical to those available from MarpolBase, but functional information for each gene may differ because of the different functional annotation process they use. Not limited to *Marchantia*, the wide variety of genomic data available from these databases can be good resources for comparative genomics studies.

Co-expression Network Toolkit (CoNekT) is a platform dedicated for the visualization and analysis of expression data for selected plant species including *M. polymorpha* (Proost and Mutwil, 2018). It provides gene expression profiles and co-expression networks using publicly available RNA-Seq data obtained from SRA, and users can compare the expression profiles of orthologous genes between species. Publicly available RNAseq data collected from *M. polymorpha* grown under an array of abiotic stresses can be visualized using the Liverwort Atlas eFP browser (Table 1).

**Table 1:** Online resources and repositories for *Marchantia polymorpha*

Organism-specific genome database	
MarpolBase	Genome Database for the reference strains Tak-1/Tak-2 https://marchantia.info/
Marchantia Gene Nomenclature DB	https://marchantia.info/nomenclature/
Genome resource portals and databases for dedicated purposes	
Ensembl Plants	https://plants.ensembl.org/Marchantia_polymorpha/Info/Index
Phytozome	https://phytozome.jgi.doe.gov/pz/portal.html#!info?alias=Org_Mpolymorpha
Co-expression Network Toolkit (CoNekT)	Visualization and analysis of expression data https://evorepro.sbs.ntu.edu.sg/
Liverwort Atlas eFP Browser	Visualization of expression data http://bar.utoronto.ca/efp_marchantia/cgi-bin/efpWeb.cgi
Public sequence database	
NCBI Assembly Database	https://www.ncbi.nlm.nih.gov/assembly
NCBI Sequence Read Archive	Repository for raw sequencing data https://www.ncbi.nlm.nih.gov/sra

## Genetics

### *Marchantia polymorpha* is a powerful system for forward genetic analysis

Forward genetics, which began in earnest following Muller’s demonstration that X-rays were mutagenic (Muller, 1927), provides an unbiased approach to identify genes by their mutant phenotype, and hence an inferred role in the process that is disrupted. The morphologically dominant, haploid phase of the *M. polymorpha* life cycle makes it a powerful system in which to carry out forward genetic screens to discover genes. Random mutagenesis in haploid organisms results in the expression of an aberrant phenotype directly in the mutagenized individual, making mutant screening rapid and logistically simple. Furthermore, there is no evidence for recent or ancient WGDs in the evolutionary history of *M. polymorpha* (Bowman et al., 2017), resulting in less genetic redundancy than in organisms that have undergone recent genome duplications, as observed in the moss *P. patens* (Lang et al., 2018).

Prior to the experimental induction of mutations Hans Burgeff collected spontaneous mutants in *M. polymorpha* (Burgeff, 1930; Burgeff, 1943), in a similar manner to the first mutations identified in the early days of *Drosophila* genetics in T. H. Morgan’s laboratory. For example, from 7,680 spores he identified a dozen spontaneous mutants and even constructed double mutant combinations (Burgeff, 1941; Burgeff, 1943). Many of the mutants affected air chamber formation, and he compared his “reductive” mutant phenotypes with the air chamber morphologies of other Marchantiopsida species that Goebel had proposed to be evolutionarily reduced (Goebel, 1930; Burgeff, 1941). Burgeff also reported a number of spontaneous chromosomal abnormalities (Burgeff, 1943), as did Gertraud Haupt, who further noted that diploid plants possessing both a U and a V chromosome were female, implying a dominant feminizing role for the U chromosome (Haupt, 1932).

Following Muller, Edgar Knapp, Gerhard Lorbeer, and Emil Heitz used X-rays (or UV irradiation) to induce mutations in other liverworts, *Sphaerocarpos* and *Pellia*, where they noted a conversion of female plants into male plants due to U chromosome rearrangements, thus defining a U chromosome “feminizer” locus (Knapp, 1935; Lorbeer, 1936; Knapp, 1938; Heitz, 1949)—see above. The earliest report of a forward genetic screen in *M. polymorpha* was some decades later, by Miller (Miller et al., 1962a, Miller et al., 1962b) who used X-rays to induce mutations in genes that resulted in a requirement for amino acids. A total of 1,588 gemmae were mutated with 15,000 R X-rays and 279 mutants with impaired growth were selected. These mutants were grown and then “diced” and replica plated onto different media to identify mutants that would only grow if amino acids were supplied in the growth media. Three classes of mutants were identified; mutants that required arginine for growth, mutants that required methionine, and mutants that required methionine and arginine. The similarity of this suite of phenotypes with phenotypes observed in bacteria that were amino acid auxotrophs allowed the authors to conclude that the ornithine cycle and the methionine biosynthetic pathway were at least partially similar in plants and bacteria.

Crosses between lines of *M. polymorpha* are simple to perform due to its dioicious nature, i.e., sperm can be readily collected from male plants and applied to female plants with no need for emasculation. However, both male and female plants must be at their reproductive phase at the time of cross, with males maturing slightly earlier than females given the same environmental conditions. Since fertile sperm of *M. polymorpha* can be cryopreserved, crosses can be performed as long as reproductive female plants are available (Togawa et al., 2018). Also, due to their dioicy, males and females collected from the same location may be polymorphic, and resulting offspring will segregate at polymorphic sites, which might affect following analyses (Ishizaki et al., 2016). To avoid this problem, pairs of wild-type male and female lines which share autosomes can be established by repeated backcrossing.

Particle bombardment was the first transformation protocol for *M. polymorpha* (Takenaka et al., 2000), and led to generation of tagged mutant lines. One of the first reported mutants identified in a forward genetic screen was *bonobo*, where reproductive structures are constitutively induced (Yamaoka et al., 2004). While the mutation in Mp*BONOBO* was not linked to the introduced plasmid, the gene, encoding a basic Helix-Loop-Helix (bHLH) transcription factor, was cloned via whole genome sequencing (Yamaoka et al., 2018). One of the *karappo* mutants described below was also isolated from transgenic lines generated by particle bombardment. The development of an efficient T-DNA transformation protocol that generates tens of thousands of transformed plants in individual transformation experiments has enabled the generation of large of T-DNA mutagenized populations for phenotypic screening (Ishizaki et al., 2008). In an independent screen *nopperabo1* (*nop1*) mutants that lack air pores and chambers were identified (Ishizaki et al., 2013b). In another independent screen for mutants with defective thallus development two mutations were identified with defective lateral organ development (Naramoto et al., 2019). The mutations resulted from T-DNA insertions into the gene encoding the MpLATERAL ORGAN SUPRESSOR1 transcription factor and suggests that the function of this family of proteins in lateral organ suppression has been independently co-opted to control lateral organ development in bryophyte gametophytes and angiosperm sporophytes. It must be cautioned, however, that as described in other systems not all mutant phenotypes will segregate with an intact T-DNA insertion, as in the case of the *karappo* mutant defective in gemma development. This mutant was identified in the same screen as *nop1*, but the T-DNA insertion was found to be not associated with the spontaneous mutation in the *KARAPPO* gene encoding a RHO PROTEIN FROM PLANTS-GUANINE EXCHANGE FACTOR (ROP-GEF) called KARAPPO (Hiwatashi et al., 2019).

The large number of T-DNA lines that can be generated by *Agrobacterium*-mediated transformation means that exhaustive screens can be carried out to identify large numbers of genes involved in particular biological processes. In a screen for genes involved in rhizoid development, 336,000 T-DNA transformed lines were screened for defective rhizoid phenotypes. This collection defined the functions of over 30 genes involved in rhizoid development. Among the proteins identified in the screen was MpROOT HAIR SIX-LIKE1 (MpRSL1) (Proust et al., 2016), an evolutionarily conserved bHLH transcription factor that positively regulates rhizoid development; two Mp*rsl1* loss-of-function and over 20 MpRSL1 gain-of-function mutants were identified in the screen. Furthermore, the screen identified four gain-of-function alleles that overexpressed the MpFEW RHIZOIDS1 (MpFRH1) miRNA, which targets the MpRSL1 mRNA for destruction (Honkanen et al., 2018). Therefore, the large scale of the mutant screen allowed the identification of both loss-of-function and gain-of-function mutations in genes encoding positive and negative regulators of rhizoid cell development. Approximately 20% of the mutations generated when plants were transformed with pCAMBIA1300 resulted in the overexpression of adjacent genes resulting in gain-of-function mutations (Honkanen et al., 2016). The opportunity to obtain both gain-of-function and loss-of-function mutations in the same gene in the same screen is a powerful tool with which to define gene function.

The value of gain-of-function mutations induced by T-DNA mutagenesis is illustrated in the search for genes that control the development of oil bodies. Kanazawa et al (2020) screened 48,500 T-DNA transformed lines for defects in oil body number by visual screening using light microscopy. They identified one mutant that developed more oil bodies than wild type. There was a T-DNA insertion 5ʹ of the Mp*ERF13* gene in this mutant. To confirm the function of Mp*ERF13* gene in oil body development, the authors generated loss-of-function mutations using CRISPR/Cas9 and these mutants developed no oil bodies. Similarly, a gain-of-function mutation in the Mp*WIP* gene developed longer rhizoids than wild type, suggesting that MpWIP is a positive regulator or rhizoid development (Jones and Dolan, 2017).

The UV radiation causes both C to T transitions and formation of thymidine dimers in DNA and has been extensively used in bacterial genetics. UV-irradiated *M. polymorpha* mutant spore populations were generated and germinating sporelings were screened for defective rhizoid phenotypes (Champion et al., 2021). The identification of multiple mutant alleles in genes encoding the MpNEK1 and the MpWAVEDAMPENED-LIKE1 (MpWDL) proteins that form defective rhizoids demonstrated the role of these proteins in microtubule organisation at the tip and the shank of the rhizoid, respectively.

Haploidy, combined with diverse mutagenesis techniques and next generation sequencing techniques and informatics pipelines make forward genetic screens feasible to identify genes involved in a diversity of processes in *M. polymorpha*. A major strength of this approach is that it allows the genetic dissection of pathways which have proved difficult to analyze using forward genetics in other model land plants. While haploid genetics brings many advantages, haploidy with a paucity of genetic redundancy also brings complications. For example, unlike similar screens in predominantly diploid organisms such as *Arabidopsis* and *Drosophila*, in haploid organisms it is difficult to perform saturation mutageneses to identify the number of loci in the genome potentially harboring lethal alleles, although in the genomic era this is not as imperative as it once was. However, for haploid organisms, lethal alleles pose a general impediment in forward genetic screens, as they may preclude the recovery of mutations in many genes of interest. Fortunately, in the age of genome editing techniques are now available to create conditional lethal alleles to circumvent this problem, albeit in a targeted manner rather than in an unbiased random mutagenesis.

### Reverse genetic approaches

*Agrobacterium*-mediated transformation is the key tool to performing reverse genetics in *M. polymorpha.* Protocols using sporelings (Ishizaki et al., 2008) and regenerating thalli (Kubota et al., 2013) have been established and widely used in multiple studies. In short, sporeling transformations are suitable for generation of a large number of transgenics (*N* > 500), while regenerating thallus transformation is used to obtain dozens of transgenics in specific genetic backgrounds. It should be noted that spores are prepared by crossing male and female plants and thus are not genetically homogeneous if the parents have different genetic backgrounds, such as the widely used laboratory lines Takaragaike-1 (Tak-1, male) and Takaragaike-2 (Tak-2, female; Chiyoda et al., 2008). To avoid genetic variation in spores, an isogenic pair of male and female lines are being established from a collection of recombinant inbred lines (Takayuki Kohchi, personal communication). Extensive descriptions and proper use of each protocol have been previously summarized (Ishizaki et al., 2016). A modified protocols, AgarTrap, enables co-culture and selection in a single Petri dish and relies on controlled humidity conditions (Tsuboyama and Kodama, 2018). It should be also noted that *Agrobacterium*-mediated transformation is applicable to transient expression assays for protein co-localization and interaction in assimilatory filaments and rhizoids of thalli (Iwakawa et al., 2021).

Four antibiotic/herbicide compounds are used for selection of sporeling transformants: hygromycin, gentamicin, chlorsulfuron, and G418 (Ishizaki et al., 2008; Ishizaki et al., 2015). Meanwhile, regenerating thallus (Kubota et al., 2013) transformation has only been successful using hygromycin and chlorsulfuron. Co-transformation of multiple constructs can be achieved in a single experiment by adding independent *Agrobacterium* cultures carrying different vectors in the co-cultivation step, and it has been estimated that ∼30% of sporelings grown on a single antibiotic plate were resistant to the second marker after infection with two *Agrobacterium* cultures in a stepwise selection experiment (Ishizaki et al., 2015). This high transformation frequency potentially allows expression of more than two transgenes in a single plant by transforming two vectors with the same selectable marker or even a marker-less vector as a second one to save markers for further transformation.

Plastid transformation in *M. polymorpha* has been established with a combination of rapidly proliferating sporelings and particle bombardment (Chiyoda et al., 2007; Chiyoda et al., 2021). In general, multiple rounds of selective steps are required to obtain homoplasmic plastid transformants in other plant species, but only a primary selection is sufficient for *M. polymorpha*, providing opportunities for functional analyses of plastid-encoded genes (Ueda et al., 2012; Ueda et al., 2014) including the development of a plastid reporter system (Boehm et al., 2016).

Binary vector series that are based on the Gateway technology have been constructed for all the four antibiotic/herbicide markers, allowing fusions of promoters and/or coding sequences (CDSs) to fluorescent protein tags, constitutive expression promoters, epitope tags, expression reporters, etc. (Ishizaki et al., 2015; Mano et al., 2018). Recently, the OpenPlant toolkit, a GoldenGate type-IIS restriction enzymes-based “Loop assembly” system for nuclear and chloroplast transformation, has been developed (Sauret-Güeto et al., 2020). The toolkit also includes a large array of tested “Level 0 DNA parts” (Sauret-Güeto et al., 2020), and is also compatible with other Phytobricks (Engler et al., 2014). In conjunction, these tools allow combinatorial assembly of various DNA elements such as promoters, CDSs, and terminators, which in turn can be combined to make vectors containing multiple expression cassettes in the same plasmid, reducing the necessity of sequential transformation or co-transformation with different selectable markers (Pollak et al., 2019).

There are three systems for induction of transient and long-term gene expression/function: a heat-shock promoter-driven transcriptional induction system for transient expression (Nishihama et al., 2016); an estradiol-inducible transcription system utilizing the chimeric artificial transcription factor, XVE, which is composed of the bacterial repressor Lex (X), the acidic transactivating domain of VP16 (V), and the regulatory region of human estrogen receptor (E) (Zuo et al., 2000; Flores-Sandoval et al., 2016). A dexamethasone-inducible protein nuclear translocation system has been also used for nuclear functioning proteins (Ishizaki et al., 2015; Yamaoka et al., 2018; Yasui et al., 2019).

Binary vectors for homologous recombination-mediated gene targeting have been used to generate gene knockouts and C-terminal knockins (Ishizaki et al., 2013a; Kubota et al., 2014; Komatsu et al., 2014; Higo et al., 2018  Yamaoka et al., 2018; Kato et al., 2020). The frequency of positive targeted lines is between 1% and 4%, which allows screening by PCR. Knockin lines in particular, provide a reliable assessment of gene expression given that the in-frame fluorescent marker is expressed from the exact locus of the target gene.

Genome editing techniques have been successfully applied to *M. polymorpha*. TALEN-mediated targeted mutagenesis was reported to occur at high frequency (Kopischke et al., 2017). CRISPR–Cas9 genome editing was first reported in 2014 generating indels at a low frequency rate, by using a humanized Cas9 and forced selection of the auxin-resistant phenotype of Mp*arf1* mutants (Sugano et al., 2014). In a newer version, a codon-optimized Cas9 gene significantly improved the genome editing efficiency (up to a 100% success rate) and was integrated into the Gateway compatible vector system with a guide RNA expression cassette (Sugano and Nishihama, 2018; Sugano et al., 2018). The OpenPlant toolkit also provides an efficient cloning system for CRISPR-Cas9 (Sauret-Güeto et al., 2020). Furthermore, large genomic deletions from about 1 kb to over 10 kb can be successfully generated by expressing two guide RNAs (Sugano et al., 2018; Monte et al., 2018; Busch et al., 2019) or by using a Cas9 nickase with four guide RNAs (Hisanaga et al., 2019a; Koide et al., 2020), with the latter considerably reducing off-target cleavage. Higher efficiency “knockin” strategies and marker-free genome editing are desirable technologies to develop.

Haploid dominancy in the life cycle of *M. polymorpha* facilitates genetic analysis, but conversely limits the chance to isolate mutants of essential genes. Artificial microRNAs (amiRs) based on endogenous MIR precursors (Flores-Sandoval et al., 2018a) recapitulate CRISPR/Cas9 generated knockout phenotypes but circumvent lethality by virtue of their controlled spatio-temporal expression or inducible expression (Flores-Sandoval et al., 2016). Recently, a conditional gene knockout strategy was developed (Sugano et al., 2018), by simultaneous co-expression of a CRISPR/Cas9 genome editing construct and a complementation transgene that is conditionally removable by a Cre-*lox*P mediated site-specific recombination (Nishihama et al., 2016). In this system, a guide RNA can be designed at an exon–intron junction so that an unmodified cDNA fragment can be directly used for the complementation (Sugano et al., 2018). Alternatively, synonymous mutations could be introduced into the target sequence of the complementary transgene to make it resistant to CRISPR-Cas9-mediated gene editing (Flores-Sandoval et al., 2018a; Monte et al., 2019; Kato et al., 2020). The Cre-*lox*P mediated conditional gene expression/deletion system was also applied to clonal analysis for investigation of cell lineages in gemmaling development using an excisable fluorescent reporter (Suzuki et al., 2020), paving the way for mosaic and cell autonomy analyses in future gene characterization studies. To complement presently available tools, development of more robust inducible systems for both gain- and loss-of-function studies is required.

As the use of molecular genetic tools described above expands, the number of transgenic and mutant lines grows rapidly. It is thus important to securely store such lines for backup, use after temporary interruption, and distribution. Although appropriately desiccated spores of *M. polymorpha* can be stored stably for years in freezers (Nakazato et al., 1999), crossing is required to prepare spores, and, as described above, the resultant progenies are genetically heterogeneous unless isogenic male and female lines are crossed. Thalli and gemmae can be clonally maintained and stored on aseptic culture media without significant reduction in viability for several weeks, even for months at 4°C under darkness. Gemmae can be stored at 4°C under darkness for years, and a vitrification-based method which enables reliable, long-term preservation of the tissue in liquid nitrogen is available (Tanaka et al., 2016a). Furthermore, sperm can be also preserved in liquid nitrogen and used for fertilization (Togawa et al., 2018).

### Tools for cell biology

Genome information and molecular genetic toolkits also facilitated studies of the cytoskeleton and cellular organelles in *M. polymorpha*, revealing unique aspects of intracellular dynamics. In these studies, probes for cytoskeletons and organelles were established, some of which are listed in Table 2. In *M*. *polymorpha*, as in other plant species, microtubules participate in various cellular processes, including cytokinesis and cell growth, and have been visualized using several fluorescent markers (Buschmann et al., 2016; Buschmann and Zachgo, 2016; Kanazawa et al., 2020). Distinct from angiosperms, bryophytes, lycophytes, ferns, and some gymnosperms have motile sperm cells as male gametes, which also comprise characteristic microtubule-containing structures. A spermatozoid comprises two flagella extending from the apical region of the cell body, which contains an axoneme with a distinctive 9 + 2 microtubule structure (Hodges et al., 2012). At the base of the flagella, basal bodies are attached to the plant-specific multilayered structure (MLS). The uppermost layer of the MLS is a microtubule array termed the spline, which extends from the MLS along the thin helical nucleus in the cell body (Carothers and Kreitner, 1968). For the actin filaments in thallus cells, a unique sliding movement affected by microtubule integrity was observed, the physiological significance of which remains unclear (Era et al., 2009; Era et al., 2013). Molecules interacting with cytoskeletons during cell growth have also been identified; a class XI myosin (MpXI), MpNEK1 (Never in mitosis A-related kinase), and MpWDL (WAVE DAMPENED2-LIKE), which act on actin filaments or microtubules, are required for normal rhizoid formation (Honkanen et al., 2016; Otani et al., 2018; Champion et al., 2021).

**Table 2:** Live-imaging tools for *M*. *polymorpha* studies

Localization	Marker	XFP	Accession (ver3.1/ver5.1)	Refs.
Actin filament	Lifeact	Venus	NA	Era et al., 2009
	mTalin	Citrine	NA	Kimura and Kodama, 2016
Microtubule	MpTUB1	GFP	Mapoly0109s0019/Mp2g16780	Buschmann et al., 2016
	MpTUB2	Citrine, TagRFP, mCitrine	Mapoly0158s0010/Mp2g09390	Otani et al., 2018; Kanazawa et al., 2020
	EB1	GFP	NA	Buschmann et al., 2016
Nucleus/Nuclear envelope	NLS	tdTomato	NA	Ishizaki et al., 2015
	H2B	tdTomato	NA	Sakamoto et al., 2022
	MpSUN	GFP	Mapoly0147s0033/Mp5g02400	Hisanaga et al., 2021
ER	GFP-HDEL	GFP	NA	Ishizaki et al., 2015
	MpSEC22	Citrine	Mapoly0023s0071/Mp2g11050	Kanazawa et al., 2016
Golgi apparatus	ST	Venus, mRFP	NA	(Kanazawa et al., 2016
	MpGOS11	mCitrine	Mapoly0001s0245/Mp1g19070	Minamino et al., 2017
	MpRRT1	TagRFP	Mapoly0033s0138/Mp1g15230	Wachananawat et al., 2020
TGN	MpSYP6A	Citrine, mRFP, mCitrine	Mapoly0140s0004/Mp3g18380	Kanazawa et al., 2016 Kanazawa et al., 2020
	MpSYP4	mCitrine	Mapoly0042s0041/Mp2g14140	Kanazawa et al., 2020
Endosome	MpRAB5	mCitrine mRFP	Mapoly0036s0134/ Mp1g08940	Minamino et al., 2017 Minamino et al., 2018
Vacuolar membrane	MpSYP2	mCitrine	Mapoly0187s0013/Mp8g15260	Kanazawa et al., 2016
	MpVAMP71	mCitrine	Mapoly0064s0109/Mp8g00880	Minamino et al., 2017
Oil body membrane	MpSYP12B	mCitrine	Mapoly0101s0013/Mp4g20670	Kanazawa et al., 2016
	MpABCG1	mCitrine	Mapoly0083s0014/Mp8g13070	Kanazawa et al., 2020
Plasma membrane	MpSYP13B	mCitrine mTurquoise2	Mapoly0055s0091/Mp2g19600	Kanazawa et al., 2016 Suzuki et al., 2020
	LTI6b	mScarletI, eGFP	AT3G05890	Sauret-Güeto et al., 2020
Plasma membrane/oil body membrane	MpPIP2	mCitrine	Mapoly0041s0003/Mp4g17210	Kanazawa et al., 2020
Extracellular space/oil body lumen	sec-mRFP	mRFP	NA	Kanazawa et al., 2020
Clathrin-coated vesicle	MpCLC1	mCitrine	Mapoly0075s0069/Mp2g03080	Kanazawa et al., 2020
Autophagosome	MpATG8a	mCitrine	Mapoly0001s0494/Mp1g21590	Norizuki et al., 2019
Plastid	MpSIG2^a^	Citrine	Mapoly0214s0004/Mp4g13380	Kanazawa et al., 2013
Mitochondrion	MpFIS1^b^	Citrine	Mapoly0147s0019/Mp5g02260	Nagaoka et al., 2017
	F1-ATPase gamma^c^	Citrine	Mapoly0062s0085/Mp7g04400	Norizuki et al., 2022
	MpIDH1	mCitrine	Mapoly0029s0048/Mp1g01980	Norizuki et al., 2022
Peroxisome	PTS1	Citrine, mRFP	NA	Mano et al., 2018

a Amino-terminus (including the transit peptide).

b A part of fluorescently tagged MpFIS1 is also targeted to peroxisomes.

c Amino-terminus (including the presequence).

**Figure 3:**
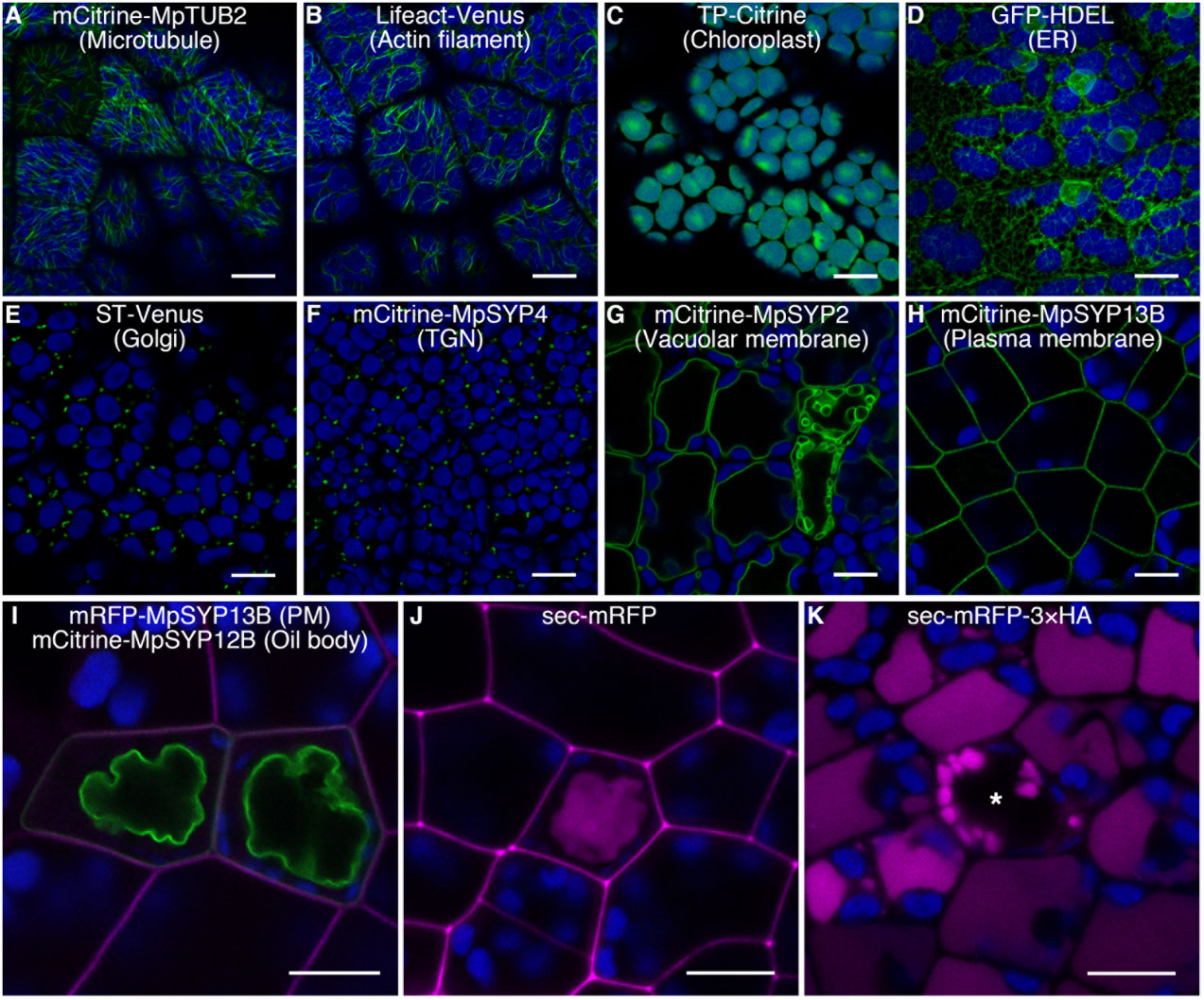
Cytoskeleton and organelles in *M*. *polymorpha* cells. A and B, Microtubules and actin filaments in *M. polymorpha* thallus cells visualized with mCitrine–MpTUB2 (A) and Lifeact–Venus(B), respectively. C, Citrine tagged with the transit peptide of MpSIG2 (TP) is targeted to chloroplasts. D, The endoplasmic reticulum (ER) visualized with mGFP–HDEL. E and F, The Golgi apparatus and *trans*-Golgi network (TGN) visualized with ST–Venus and mCitrine–MpSYP4, respectively. G and H, Thevacuolar membrane and the plasma membrane (PM) visualized with mCitrine–MpSYP2 and mCitrine–MpSYP13B, respectively. I, Oil body cells expressing mRFP–MpSYP13B and mCitrine–MpSYP12Bunder their own promoters, which target the PMs and the oil body membrane, respectively. J and K, *M. polymorpha* thallus cells expressing sec–mRFP (J), or sec–mRFP–3×HA (K) under the constitutiveMp*SYP2* promoter. sec–mRFP is transported to the extracellular space and oil body lumen in non-oil body and oil body cells, respectively, whereas it is mistargeted to the vacuolar lumen when tagged with 3×HA at the C-terminus. An asterisk in K indicates the oil body. Green, magenta, and blue pseudocolor indicate the fluorescence from mGFP or YFP (mCitrine, Venus, or Citrine), mRFP, and chlorophyll, respectively. Bars = 10 μm.

A series of fluorescent markers have been developed to visualize organelles in living cells (Table 2 and Figure 3). Endomembrane organelles are interconnected by the membrane trafficking system, which involves evolutionarily conserved machinery, such as soluble N-ethylmaleimide sensitive factor attachment protein receptor (SNARE) proteins and RAB GTPases. Each SNARE protein or GTPase localizes to one or more specific organelles and is thus used as a specific organelle marker (Kanazawa et al., 2016; Kanazawa and Ueda, 2017; Minamino et al., 2018). SNARE proteins also travel in cells along membrane trafficking pathways; therefore, they are also used as cargo models in studies of membrane trafficking.

For example, rapid endocytic transport and degradation of plasma membrane SNAREs were detected during the dynamic reorganization of organelles in *M*. *polymorpha* spermiogenesis (Minamino et al., 2017; Minamino et al., 2022). Multimodal autophagic degradation of cytoplasmic components is also potentiated during spermiogenesis (Norizuki et al., 2022). Thus, spermiogenesis in *M. polymorpha* involves multiple distinct degradation pathways and occurs in a cell-autonomous manner, differing from spermiogenesis in mammalian cells, which requires phagocytic elimination of excess cytoplasm by neighboring Sertoli cells (O'Donnell et al., 2011). The plasma membrane SNARE MpSYP13B was also shown to target the haustorium-like structure formed upon oomycete infection (Carella et al., 2018), and homologous MpSYP12B targets the oil body membrane (Kanazawa et al., 2020; see also the section “Oil bodies”), further confirming the utility of these proteins as organelle markers and/or cargo.

Live imaging using fluorescent markers is a powerful methodology in plant cell biology, but several difficulties remain. Green plants, including *M*. *polymorpha*, possess autofluorescent pigments, such as chlorophyll and flavonoids (Berland et al., 2019). To distinguish autofluorescence from signals produced by products of interest, time gating and/or spectral imaging should be employed (Kanazawa et al., 2016; Kodama, 2016). The effects of added tags should also be considered. Fluorescent proteins frequently possess oligomerization activity, which can alter the organelle morphology or the efficiency of Förster/fluorescence resonance energy transfer (FRET) (Zacharias et al., 2002; Segami et al., 2014; Kanazawa et al., 2016). Monomeric versions of fluorescent proteins generated by introducing monomerizing mutations, such as the A206K mutation in the green fluorescent protein and its derivatives (Zacharias et al., 2002), should be selected for marker construction. Additionally, exogenously added tags can act as unintended targeting/localization signals. For example, the 3 × human influenza hemagglutinin epitope (3 × HA) acts as a vacuolar sorting signal in thallus cells of *M. polymorpha* (T. Ueda and T. Kanazawa, unpublished data; Figure 3).

## Vignettes of recent research highlights of the post-genomic age

Given the burgeoning quantity of literature, in this last section we provide a few vignettes highlighting how utilizing *M. polymorpha* as a model system has contributed to furthering our understanding of several diverse biological phenomena. From these examples, it is hoped the reader will be inspired to contemplate whether incorporating *M. polymorpha* as a model may advance their own research program. Due to the extensive literature on each of these topics, the references listed are restricted to recent *M. polymorpha* studies.

### Reproductive biology

Only a brief overview highlighting some significant recent papers is presented as the topic has been recently reviewed in detail (Hisanaga et al., 2019b; Kohchi et al., 2021). Sex is determined in the gametophyte generation and the gametangiophores, a modified thallus, are initiated following a bifurcation of the shoot meristem. The size of the meristem, i.e. the apical cell and its immediate derivatives is modulated by the activity of a CLE peptide signaling system, but in an opposite manner than the homologous CLV3-based signaling system operates in the shoot meristem of angiosperms, and without the involvement of downstream WOX activity (Hirakawa et al., 2020).

**Figure 4:**
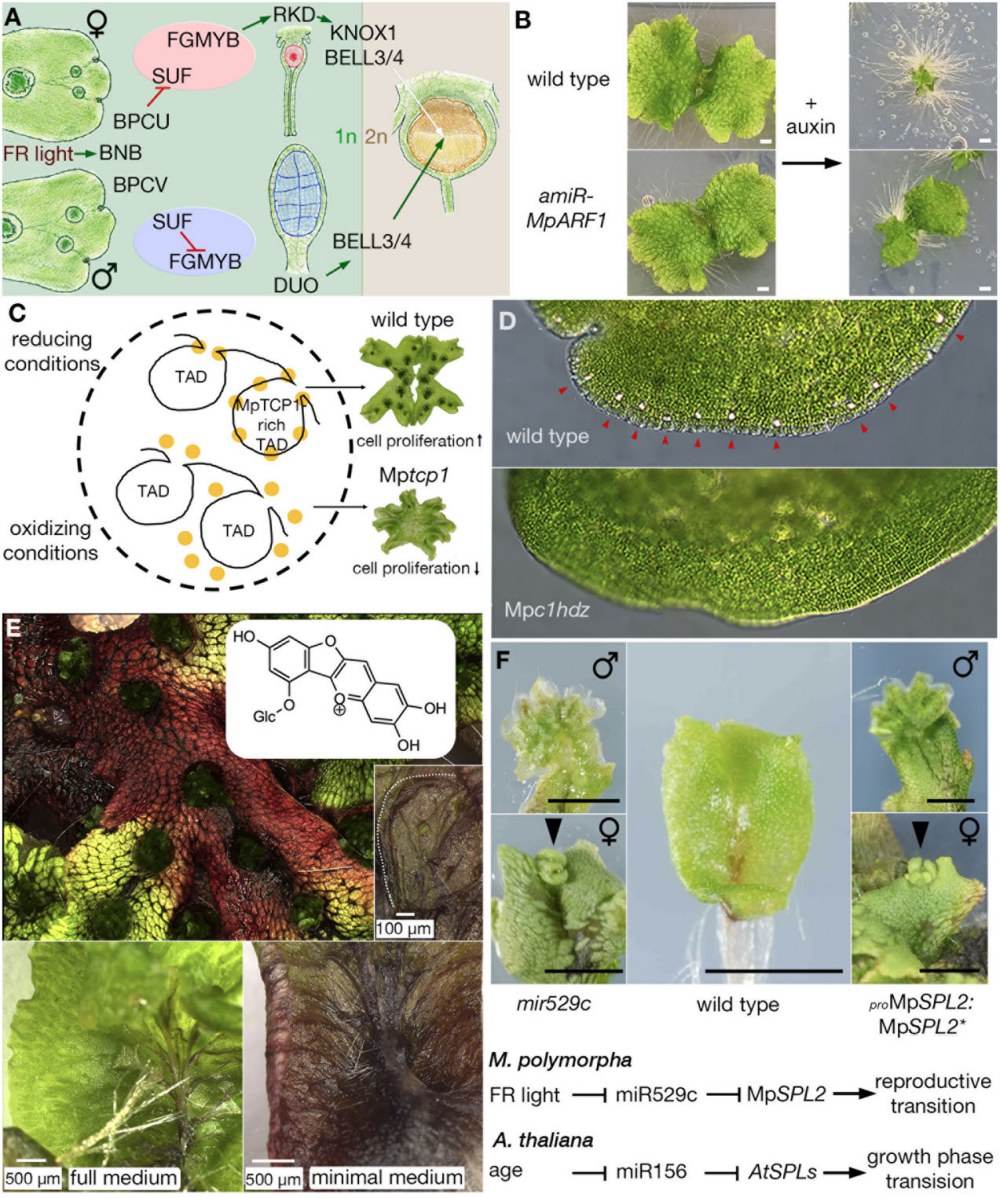
Pictorial depictions of some research highlights. A, Outline of the genetic regulation of reproduction in *M. polymorpha*, with the haploid gametophyte (left) and diploid sporophyte (right). Arrows represent promotion and bars represent repression within the genetic networks (as outlined in the text); some interactions are known to be direct, while others may be indirect. Sex is determined by the distinct regulation of the FGMYB-SUF module (red and blue ovals for female and male, respectively), by BPCU carried by the U chromosome in females. B, Growth of wild-type gemmalings in the presence of exogenous auxin leads to suppression of growth and ectopic rhizoid formation. In contrast, reduction of Mp*ARF1* function, as in this line in which an artificial microRNA targeting Mp*ARF1* transcripts is constitutively expressed, results in loss of auxin sensitivity. C, Model for redox control of MpTCP1 activity in the nucleus. Under reducing conditions, MpTCP1 (yellow dots) binds to TAD boundaries and to genomic regions in MpTCP1-rich TADs. In contrast, oxidization causes dissociation of MpTCP1 from the DNA consequently altering gene expression, leading to cell proliferation repression and reduced thallus growth. D, Oil body cells (the bright cells at the arrowheads) are conspicuous near the gemma margin in wild type, but are lacking in Mp*c1hdz* gemmae. The formation of a toothed marginal cells is also lacking in Mp*c1dhz* mutants whose margins are smooth. E, Nutrient deprivation induces production of the cell wall localized red pigment auronidin. Ventral and dorsal views are shown in the upper and lower panels, respectively. The ventral image is of a plant on minimal medium. The upper right image shows a close up off a single scale, with the scale edge indicated. Photos courtesy of Nick Albert and Yanfei Zhou. F, The miR529c-Mp*SPL2* module regulates sexual reproductive development. While the vegetative to reproductive transition in wild-type requires a FR light stimulus, either loss-of-function Mp*mir529c* alleles and gain-of-function miR529-resistant Mp*SPL2* (MpSPL2*) developed antheridiophores or archegoniophores in males and female, respectively, in the absence of FR light stimulus. Arrowheads indicate archegoniophores formed at the apical region. The putative gene regulatory pathways in *M. polymorpha* and *A. thaliana* are presented at lower right. Scale bars: 5 mm.

*Marchantia polymorpha* is a long day plant (Wann, 1925), with reduced R:FR light conditions required for the reproductive transition (Inoue et al., 2019). The molecular machinery for R/FR light perception and the downstream processes in *M. polymorpha* are similar to that observed in angiosperms, except only a single phytochrome and *PHYTOCHROME-INTERACTING FACTOR* (Mp*PIF*) are present (Nishihama et al., 2015; Inoue et al., 2016; Inoue et al., 2019). As in Arabidopsis, orthologues of GIGANTEA (Mp*GI*) and FLAVIN-BINDING KELCH REPEAT F-BOX1 (Mp*FKF*) mediate the long day-dependent transition (Kubota et al., 2014). Similar to SQUAMOSA PROMOTER-BINDING LIKE (SPL) transcription factors mediating phase transitions in angiosperms, Mp*SPL2* promotes the reproductive transition while miR529 represses the transition by targeting Mp*SPL2* transcripts (Tsuzuki et al., 2019). A bHLH transcription factor, BONOBO, is necessary and sufficient for gametangial development and generative cell specification (Yamaoka et al., 2004, 2018; Figure 4, A). Identification of BONOBO in *M. polymorpha* facilitated understanding of the role of orthologous genes in Arabidopsis that mediate male germ cell specification (Yamaoka et al., 2018). Downregulation of an AUXIN RESPONSE FACTOR encoded by Mp*ARF3* is also necessary for the reproductive transition to occur although its precise role is as yet unknown (Flores-Sandoval et al., 2018a). The sex of gametangiophores and gametangia is mediated by the regulation of the autosomal complex locus FGMYB/SUF by the U-chromosome linked feminizer, as described previously (Hisanaga et al., 2019a; Iwasaki et al., 2021).

Similar to the role of the MYB transcription factor *DUO1* in promoting sperm cell differentiation in angiosperm pollen, within developing antheridia Mp*DUO1* is also required for proper sperm differentiation but its downstream targets have diverged (Higo et al., 2018). The origin of specific domains within DUO1 in a charophyte algal ancestor was a defining event in the evolution of sperm within this lineage (Higo et al., 2018). Within the archegonia, an RWP-RK transcription factor, MpRKD, is required for proper egg cell development (Koi et al., 2016; Rövekamp et al., 2016). This parallels a similar role for orthologous genes in angiosperms. Mp*RKD* is also expressed in the antheridia and appears to have a role in the development of both female and male gametes as well as roles during vegetative growth of the gametophyte (Koi et al., 2016; Rövekamp et al., 2016). Following fertilization, gamete expression of TALE-homeodomain proteins of the KNOX and BELL subclasses is required to initiate the diploid sporophyte genetic program (Dierschke et al., 2021; Hisanaga et al., 2021). Maternal MpKNOX1 is absolutely required while both paternal and maternal MpBELL contributes to initiation of the sporophyte generation, with the MpKNOX1-MpBELL heterodimers also likely active during early sporophyte development (Dierschke et al., 2021; Hisanaga et al., 2021).

### Hormone biology

While many of the latest advances have been reviewed recently (Bowman et al., 2019a), we provide a brief overview and highlight some significant recent papers. Studies in *M. polymorpha* have been pivotal in ascertaining how the auxin transcriptional response pathway was assembled from both neofunctionalization of pre-existing genes combined with evolution and diversification of new paralogs (Eklund et al., 2015; Flores-Sandoval et al., 2015; Kato et al., 2015; Bowman et al., 2017; Kato et al., 2017; Flores-Sandoval et al., 2018a; Flores-Sandoval et al., 2018b; Mutte et al., 2018; Kato et al., 2020; Figure 4, B). Similar to auxin, the canonical land plant jasmonic acid pathway was assembled from both neofunctionalization of pre-existing genes and addition of new components, with the pathway acting in a similar manner to that characterized in seed plants (Monte et al., 2018; Monte et al., 2019; Peñuelas et al., 2019). The major difference is that the receptor, COI1, in *M. polymorpha* perceives dinor-12-oxo-10,15(Z)-phytodienoic acid (dn-OPDA) rather than jasmonoyl-isoleucine, as in Arabidopsis, due to a single amino acid difference in the receptor (Monte et al., 2018). As in other land plants, the pathway is involved in response to pathogens (Gimenez-Ibanez et al., 2019; Matsui et al., 2020). Of great interest is that dn-OPDA regulates thermotolerance gene expression in an COI1-independent manner in both *M. polymorpha* and the charophyte alga *Klebsormidium nitens*, suggesting the co-option of this molecule from an ancestral (and retained) function into the COI1-mediated signaling pathway (Monte et al., 2020).

The canonical land plant cytokinin signaling pathway is conserved in *M. polymorpha*, with its disruption affecting thallus growth and cell proliferation (Flores-Sandoval et al., 2016; Aki et al., 2019). While *M. polymorpha* lacks the genetic machinery to respond to mycorrhizal fungi and also some strigolactone biosynthetic genes, the signaling pathway characterized as downstream of strigolactone/karrikan in seed plants is present and is required for proper thallus development and dark-induced gemma dormancy (Mizuno et al., 2021). Consistent with this pathway having an ancestral function in plant–mycorrhizal interactions, strigolactone signaling is required for establishment of arbuscular mycorrhizal interactions with *Marchantia paleacea* (Kodama et al., 2021). As with other archegoniate plants, *M. polymorpha* lacks a characterized ethylene forming enzyme, but again the signaling pathway typically downstream of ethylene is present and acts in gemma dormancy and thallus growth (Li et al., 2020a; Katayose et al., 2021). Similarly, while *M. polymorpha* does not appear to produce canonical seed plant gibberellic acid isoforms, components of the downstream signaling pathway, including the DELLA GRAS transcription factor are present, with the latter acting in the coordination with growth in response to environmental stressors (Hernández-García et al., 2021).

A key phytohormone involved in response to stressors of the terrestrial environment is abscisic acid (ABA), with a canonical land plant biosynthesis and response pathway present and functional in *M. polymorpha* (Tougane et al., 2010; Bowman et al., 2017; Eklund et al., 2018; Jahan et al., 2019). The canonical land plant ABA response pathway was assembled from pre-existing components present in the ancestral alga (Bowman et al., 2017; de Vries et al., 2018), and intriguingly, the incorporation of ABA into a pre-existing stress response pathway acts as a rheostat, with ABA modulating a basal ligand-independent receptor activity (Sun et al., 2019).

### Cell biology

As alluded to previously, studies in *M. polymorpha* have contributed to the elucidation of ancestral genetic programs directing rhizoid tip growth and other epidermal outgrowths (Honkanen et al., 2016; Proust et al., 2016; Honkanen et al., 2018; Champion et al., 2021). While the life history of *Marchantia* does not provide a prolonged developmental stage as accessible for cell biological observations as does the protonemal growth that occurs in mosses, studies have contributed to advances in other aspects of cell biology. For example, in *Marchantia* cell division is similar to vascular plants, however, when preparing for mitosis this liverwort forms centrosome-like organelles, termed “polar organizers”. These microtubule-organizing structures are believed to represent an evolutionary “left-over” from algae and may have a function in controlling the polarity of cell division (Buschmann et al., 2016; Buschmann and Zachgo, 2016).

Reactive oxygen species (ROS) have long been known for their roles in mediating stress response and their regulatory activities controlling the balance between cell proliferation and differentiation processes were recently recognized, with H_2_O_2_ promoting cell differentiation, and $O_{2}^{-}$ promoting stem cell maintenance in Arabidopsis (Zeng et al., 2017). In *M. polymorpha*, the MpTCP1 transcription factor is expressed in the apical notch. Mp*tcp1* gemmalings exhibit a reduced cell proliferation resulting in smaller thallus formation and production of increased H_2_O_2_ levels. Notably, MpTCP1 proteins bind redox-dependently to DNA mediated by the highly conserved cysteine C131. This enables MpTCP1 proteins to sense intracellular redox-changes and to respond by regulating during vegetative growth a complex redox-network comprising ROS producing and metabolizing enzymes (Busch et al., 2019). MpTCP1proteins are enriched at the boundaries of self-interacting genomic TAD regions that flank interstitial heterochromatin, where, however, MpTCP1 activity seems to be dispensable (Karaaslan et al., 2020). Under oxidizing conditions, MpTCP1 binding to TCP1-rich TADs, which might represent a novel type of redox-sensing nuclear microcompartment, is abolished, affecting ROS levels and thallus growth (Figure 4, C). Redox-sensitive DNA-binding was also demonstrated for the bZIP TF MpTGA expressed in apical notches (Gutsche et al., 2017). *Marchantia polymorpha* possesses genes encoding flavodiiron proteins (FLV), widespread enzymes that suppress the production of ROS. FLVs limit photooxidative damage of plants living in aqueous environments, whereas angiosperms lost FLV genes and their activities were likely compensated by diversification of alternative ROS protective mechanisms (Shimakawa et al., 2017). These observations strengthen the importance of sensing and responding to ROS changes together a diversification of ROS processes during land plant terrestrialization.

### Oil bodies

The post-genomic era not only provided new insights on conserved features among all land plants but also on liverwort synapomorphies such as oil bodies. In *M. polymorpha* oil bodies can be observed as a single brownish organelle present in scattered idioblastic cells (Shimamura, 2016). Early work noted that the fragrance of the crushed plant tissues correlates with the prevalence of oil bodies (Gottsche, 1842; Lindberg, 1862). Sesquiterpenes and *bis*-benzyl acids are the main components of essential oils in liverworts (Asakawa et al., 1979). More recently, it was shown that sesquiterpenoids can be isolated directly from oil bodies, while remaining undetectable in parenchymatic cells (Tanaka et al., 2016b). The compounds isolated from oil bodies are highly toxic for the plant itself (Tanaka et al., 2016b), suggesting that oil bodies are required to accumulate these compounds and avoid self-cytotoxicity. Oil bodies are similar in this function to other secretory structures like glandular trichomes in angiosperms, but that have evolved independently (Lange, 2015).

These organelles may have helped liverworts to develop an enormous and unique chemical diversity (Chen et al., 2018; Asakawa and Ludwiczuk, 2018). Isoprene synthases, responsibly for the biosynthesis of sesquiterpenoids, localize in the oil body membrane (Suire et al., 2000). Furthermore, these terpene synthases belong to lineages of fungal-type terpene synthases that were acquired via horizontal gene transfer and that are absent from angiosperms (Jia et al., 2016; Kumar et al., 2016; Takizawa et al., 2021). Less is known about the biosynthesis of *bis*-bibenzyls (Friederich et al., 1999) and other oil body-specific compounds such as cannabinoid-like compounds found in some *Radula* species (Hussain et al., 2018).

The oil bodies from liverworts are often confounded with lipid droplets present in most land plant species, particularly in seeds (Lundquist et al., 2020), despite having distinct cellular characteristics. Moreover, lipid droplets are also observed in liverworts, and they do not fuse with oil bodies (Duckett and Ligrone, 1995). Unlike lipid droplets that are surrounded by a single layer of lipids, oil bodies are surrounded by a bilayer membrane with similar properties to the plasma membrane (Kanazawa et al., 2020). A SNARE protein, MpSYNTAXIN OF PLANTS 12B (SYP12B), is specifically localized to the oil body membrane (Kanazawa et al., 2016), and as it is a close paralog of the syntaxin involved in cell plate formation, it suggests the oil body is formed in a manner analogous to cell plate formation, a novel mechanism to form a new cellular organelle. Consistent with this hypothesis, redirection of secretory pathways is important for the oil body formation (Kanazawa et al., 2020). Thus, examination of the molecular events of oil body formation in liverwort species with multiple oil bodies per cell, as in the Jungermanniopsida, is of interest.

A long-debated question is the biological function of oil bodies. Different functions have been proposed including nutrient storage, or biotic and abiotic stress protection. However, the most compelling evidence is associated with defense against herbivores. In 1888, feeding experiments with snails suggested snails prefer to eat liverworts after being leached with alcohol (Stahl, 1888). Liverwort fossils from the Middle Devonian also suggest that herbivores avoid chewing oil body cells (Labandeira et al., 2014), supporting oil body are an ancient strategy as deterrent against herbivores. More recently, genetic experiments permitted to demonstrate more clearly the biological function of oil bodies, at least for *M. polymorpha*. Two transcription factors, MpC1HDZ and MpERF13, were found to be important for oil body cell differentiation (Figure 4, D). Feeding experiments with pill-bugs showed that mutant plants lacking oil bodies are preferred (Kanazawa et al., 2020; Romani et al., 2020). As expected, these plants are also strongly reduced in sesquiterpenoids (Romani et al., 2020). In a similar way, mutant plants reduced in terpenoids are more susceptible to the herbivory of *Spodoptera littoralis* (Peñuelas et al., 2019), emphasizing the link between chemical content and biological function. The antibiotic activity of plant extracts against bacteria and fungi were also diminished in mutants reduced in oil body-specific compounds (Wu et al., 2018; Romani et al., 2020), but it remains unknown whether this has any effect in the context of biological interactions. In summary, oil bodies are a unique feature of liverworts that have gained great attention in recent years, showing novel insights for cell biology but also promise for biochemistry and biotechnology applications.

### Pigmentation

The major plant pigments are the chlorophylls, carotenoids, and flavonoids. The core carotenoid biosynthetic pathway is conserved across land plants, albeit with variations in enzyme characteristics within the different lineages (Takemura et al., 2015). As yet, there is only limited data regarding chlorophyll biosynthesis in *M. polymorpha* (Ueda et al., 2014). The majority of studies have been on the flavonoids, which are biosynthesized by a branch of the phenylpropanoid pathway. The flavonoid pathway is generally regarded as specific to land plants, with potential parallel evolution in fungi (Bu et al., 2020). In angiosperms, important flavonoid groups include the anthocyanin pigments and the colorless UVB-absorbing flavonols and flavones. Research to date on the flavonoids of *M. polymorpha* has revealed strong conservation of some pathway components with angiosperms but also notable divergence. UV-B responses mediated by the UVR8 photoreceptor are conserved between *M. polymorpha* and Arabidopsis, including production of similar types of protective flavonoids (Clayton et al., 2018; Kondou et al., 2019). The striking difference between *M. polymorpha* (and presumably other liverworts) and angiosperms is with regard to the colored flavonoids. The red pigments of angiosperms, the anthocyanins, are replaced in liverworts with a red flavonoid pigment group named “auronidins” (Kunz et al., 1994; Berland et al., 2019; Figure 4, E). Some of the environmental triggers that induce anthocyanin production in angiosperms also induce auronidin accumulation in liverworts, in particular nitrogen deficiency, strong white-light, phosphate starvation, and pathogen attack (Nagai, 1915; Albert et al., 2018; Kubo et al., 2018; Carella et al., 2019; Rico-Reséndiz et al., 2020), indicating shared functionality. However, the cell-wall-bound nature of auronidins raises the possibility for other functionalities in liverworts that would be an interesting area for future studies. For example, auronidins have been shown to reduce cell penetration by *Phytophthora palmivora* hyphae (Carella et al., 2019).

There are several outstanding questions on the biosynthesis and function of nonphotosynthetic pigments in bryophytes that studies on *M. polymorpha* can help answer. The eco-physiological functions of red pigmentation in bryophytes in general are poorly understood. Also, the biosynthetic pathway to auronidins is only partly elucidated, which gaps regarding intracellular transport, structural variety, and incorporation into the cell wall. Moreover, despite previous chemical characterization of the specialized metabolites of liverworts (Asakawa et al., 2013), there are probably many metabolite structures and functionalities to be discovered in *M. polymorpha*, as illustrated by the recent finding of novel plant aminochrome pigments (Busch et al., 2019). The evolutionary history of flavonoid biosynthesis and red pigmentation is also still a subject of debate (de Vries et al., 2017; Yonekura-Sakakibara et al., 2019; Davies et al., 2020). Finally, there are many unresolved questions on the transcription regulation of flavonoid biosynthesis (Albert et al., 2018; Kubo et al., 2018), for which the reduced diversity of transcription factor genes in *M. polymorpha* should provide a research advantage over angiosperm models.

### microRNA biology

In *M. polymorpha*, 265 miRNA families producing 129 unique mature miRNAs have been identified (Lin et al., 2016; Tsuzuki et al., 2016). As seen in other species, the majority, 256 families, are phylogenetically limited to liverworts or a subset thereof. Of the evolutionarily more ancient miRNAs families, seven (miR160, miR166, miR171, miR319/miR159, miR390, miR408, and miR529/miR156) were likely present in the ancestral land plant, one (miR536) evolved in the ancestral bryophyte, and one (miR1030) in the common ancestor of mosses and liverworts (Arazi et al., 2005; Axtell and Bowman, 2008; Lin and Bowman, 2018; Zhang et al., 2020a). The evolutionarily ancient miRNAs often have conserved targets, as exemplified by miR166 targeting C3HDZ mRNAs (Floyd and Bowman, 2004) and miR160 targeting class C ARF mRNAs (Flores-Sandoval et al., 2018a).

In other cases, the evolutionary scenarios are more complicated. For example, miR156, targets transcripts of *SPL* transcriptional factor family genes thereby suppressing precocious reproductive transitions in Arabidopsis and other seed plants. However, miR156 is not present in *M. polymorpha*, but similar miRNAs, miR529a-c, sharing the core 16/17 nucleotides with miR156 are detected, with miR529c targeting the homologous nucleotides of *SPL2* mRNA as miR156 (Tsuzuki et al., 2016; Figure 4, F). Functional analysis confirmed that miR529c is responsible for repression of Mp*SPL2* gene expression, taking the place of miR156 in other plant species (Tsuzuki et al., 2019). Thus, miR156/miR529 constitute a single miRNA family targeting SPL gene expression. Similar to its role in seed plants, the miR529c-Mp*SPL2* module regulates the reproductive transition in *M. polymorpha* (Tsuzuki et al., 2019). In *P. patens*, miR156 also controls developmental timing, but works in an opposite manner to that of seed plants and *M. polymorpha*, with *P. patens* miR156 promoting the transition from protonemata to gametophore growth (Cho et al., 2012). However, in *P. patens*, the regulatory network of miR156 is entangled with the miR390-triggered tasiRNA pathway, making the scenario more complex. Thus, conserved miRNAs may provide variations on a theme, with functional studies required for elucidation of precise function.

Functional studies about phylogenetically restricted miRNAs provide insight into lineage specific evolutionary processes. For example, the previously mentioned MpFRH1 (Mp*MIR11681*) miRNA targeting of Mp*RSL1* mRNA provides an example of an independent evolution of a spatial patterning mechanism for rhizoid initiation that appears to be conserved throughout liverworts (Proust et al., 2016; Honkanen et al., 2018).

### Epigenetics

Control of gene expression through DNA methylation is a fundamental epigenetic mechanism involved in the defense against invading nucleic acids and in the regulation of developmental pathways (Feng et al., 2010). The *M. polymorpha* genome encodes core members of the major sRNA mediated epigenetic pathway operating in plants—termed the RNA-dependent DNA methylation (RdDM) pathway—(Matzke and Mosher, 2014), including 24-nt siRNAs and two plant-specific RNA polymerases involved in gene silencing, Nuclear RNA polymerase D (POLIV) and the Nuclear RNA polymerases E (POLVa and POLVb) (Huang et al., 2015; Bowman et al., 2017; Aguilar-Cruz et al., 2019). DNA methylation has been proven important for silencing of transposable elements and for the perpetuation of cell identity during the gametophytic phase of *M. polymorpha* (Ikeda et al., 2018). Interestingly, the levels of gene body methylation (gbM), defined as the pattern of DNA methylation in the CG context located between the transcription start and the transcription termination sites of genes originally described in angiosperms (Bewick et al., 2016), has been shown to be dramatically reduced in *M. polymorpha* (Bowman et al., 2017; Schmid et al., 2018) and similar to that observed in other plant species lacking gbM (Bewick et al., 2016; Bowman et al., 2017; Bewick et al., 2017). Reprogramming of genomic DNA methylation during the life cycle of *M. polymorpha* has been documented (Schmid et al., 2018). Levels of DNA methylation are low in vegetative tissues and they are a dramatically increased in reproductive tissues. While a clear enrichment of cytosine methylation in the CG and CHG context, gene bodies, and gene flanking regions was observed in antherozoids and archegonia, gain of DNA methylation during sporophyte development was mostly limited to the CHH context, transposable elements and repeats (Schmid et al., 2018). In addition to heterochromatic 24-nt siRNAs, *trans*-acting siRNAs have been described. Mp*TAS3* has two binding sites for MpmiR390-guided cleavage that trigger the biogenesis of tasiARFs which accumulate at high levels and potentially target Mp*ARF2* (Xia et al., 2017; Flores-Sandoval et al., 2018a; Lin and Bowman, 2018). Moreover, tasiAP2 might not target an Ap2 homolog, but rather a gene encoding cytochrome P450_78 with 747 reads of degradome evidence (Lin et al., 2016).

### Synthetic biology

The ease of cultivation, short life cycle, and stable genetic transformation of *M. polymorpha*, together with other advantages as a model system extended its use not only to molecular biology and evolution studies but also to novel fields such as synthetic biology (Boehm et al., 2017). Synthetic biology applications include the production of heterologous proteins of industrial interest, the engineering of biosynthesis pathways of valuable secondary metabolites, construction of complex orthologous genetic circuits for building biosensors, among several others. The premise behind developing *Marchantia* as a synthetic biology chassis is that it could serve as a testbed to generate novel functions translatable to crop plants or in *Marchantia* itself. Vector sets such as the OpenPlant kit for *Marchantia* (Sauret-Güeto et al., 2020), are compatible with the common syntax for plant synthetic biology (Patron et al., 2015) enabling the flexible combination of standardized parts, such as coding and regulatory regions, and tags. These genetic parts, the so-called Phytobricks, can also be assembled using Type-IIS restriction enzyme cloning in a high-throughput manner using biofoundry capabilities (Cai et al., 2020).

For protein expression engineering, the chloroplast is a preferred target. For this purpose, pentatricopeptide repeat proteins (PPRs) have the ability to stabilize chloroplast mRNA and confer high expression levels. In *Marchantia*, PPRs have been using to optimize the hyper-expression, with a yield of up to ∼15% of total soluble protein obtained for expression of heterologous proteins in the chloroplast (Frangedakis et al., 2021). This combination of chloroplast transformation and PPRs set the foundation for the rapid and scalable production of valuable proteins for therapeutic use among other applications. Another recently published example is the use of *M. paleacea* as a chassis for the engineering of a heterologous biosynthetic terpenoid pathway, the production of patchoulol, obtaining a yield of up to 3.2 mg g^−1^ of *Marchantia paleacea* dry weight (Zhang et al., 2020b). These yields are still far from the required for commercial applications, but are comparable with other plant chassis such as mosses (Reski et al., 2018) and open the possibility for improvement and translating to crop species more suitable for scaling-up.

## Outlook

*Marchantia polymorpha* has served as a model organism to investigate biological processes for more than two centuries. Initially this was due to its ubiquity and ease of growth in culture. In the era of genomics *Marchantia* also served as a model for the organellar genomes, and, perhaps fortuitously, the lack of ancient whole genome duplications in the liverwort lineage has results in a nuclear genome with a paucity of genetic redundancy, facilitating both forward and reverse genetic approaches. Given its genomic attributes, phylogenetic positions, and myriad of tools available for genetic manipulation, it is envisioned that *Marchantia* will be one of the species on the Rosetta stone to better understand how land plants conquered the terrestrial environment.

## Notes

J.L.B. prepared Figures 1 and 2; T.U. and T.Ka prepared Figure 3; J.L.B., F.R., S.Z., K.M.D., K.F., M.T., Y.W. and E.F-S collectively prepared Figure 4. J.L.B. assembled text provided by all the other authors: M.A-V, F.B., J.L.B., L.N.B., P.C., A.A-C, K.M.D., T.D., L.D., A.D-A, T.J.F., E.F-S, K.F., K.I., R.J., T.Ka, H.K., T.Ko, J.L., S-SL., H.N., R.N., F.R., S.S., Y.T., M.T., T.U., Y.W., K.T.Y. and S.Z.

The author responsible for the distribution of materials integral to the findings presented in this article in accordance with the policy described in the Instructions for Authors (https://academic.oup.com/plcell) is: John Bowman (john.bowman@monash.edu)
